# Mission to Psyche: Including Undergraduates and the Public on the Journey to a Metal World

**DOI:** 10.1007/s11214-023-00967-x

**Published:** 2023-04-05

**Authors:** Catherine D. D. Bowman, Linda T. Elkins-Tanton, Adriana Talamante, James F. Bell, Ernest Cisneros, Alexandra Cook, Jason D. Frieman, Danya Gainor, Jamie Hunziker, Shaheer Khan, Christopher R. Lawler, Jessica Maschino, Timothy J. McCoy, Kaxandra Nessi, Rona Oran, David Seal, Amber Simon, Rohit Singh, Carol M. Tolbert, Karin Valentine, Benjamin Weiss, Daniel D. Wenkert, David A. Williams

**Affiliations:** 1 School of Earth and Space Exploration, Arizona State University, Tempe, AZ USA; 2 NASA Glenn Research Center, Cleveland, OH USA; 3 Smithsonian Institution, Washington, DC USA; 4 Massachusetts Institute of Technology, Cambridge, MA USA; 5 Jet Propulsion Laboratory, Pasadena, CA USA

**Keywords:** Psyche, Asteroid, Undergraduate students, Public engagement, STEAM, Interdisciplinary

## Abstract

The NASA Psyche mission’s program to engage university undergraduates and the public in the mission is inspired by and built upon the extensive foundation of public engagement, educational outreach activities, and expertise of NASA and mission partner institutions. The program leverages the enthusiasm and contributions of undergraduates nationwide to the benefit of the mission, the students and their institutions and communities, and the broader public. Psyche Student Collaborations consists of four main programs, two (Psyche Capstone and Psyche Inspired) are available solely to undergraduates enrolled at universities or community colleges in the United States and its territories and two (Innovation Toolkit free online courses and Science Outreach Interns and Docents) invite broader participation by engaging the talents and creativity of undergraduate interns to help create content and events to reach the public and lifelong learners. Together, these offerings provide multiple entry points and a spectrum of intensity of experiences, numbers of participants, disciplinary diversity, and mode of delivery. Involving undergraduates in all phases of the program supports the development of the next generation of explorers, contributes to the nation’s workforce preparation, and complements NASA’s existing undergraduate offerings by providing long-term opportunities for students to participate with the mission through established postsecondary education structures like capstone courses.

## Introduction

NASA’s Discovery Program (https://www.nasa.gov/planetarymissions/discovery.html) aims to “achieve outstanding results by launching more smaller missions using fewer resources and shorter development times” than larger flagship missions. Discovery missions are led by a principal investigator and are selected through peer review during a multi-stage selection process (NASA 2014). In January 2017, NASA selected the Psyche mission to investigate the asteroid (16) Psyche, led by Arizona State University (ASU), as its 14th Discovery mission.

Among the goals of the Discovery Program, as stated in the 2014 Discovery Announcement of Opportunity (AO), is to “announce scientific progress and results in the peer-reviewed literature, popular media, scholastic curricula, and materials that can be used to inspire and motivate students to pursue careers in science, technology, engineering, and mathematics” (NASA 2014). Although the 2014 AO precluded an *Education and Communication* (*E&C*) *Program*, the mission team submitted a plan for the optional *Student Collaboration* opportunity, defined in the AO as “a separate part of the proposed investigation” that “may not increase the mission development risk” and “must include appropriate plans for the mentoring and oversight of students to maximize the opportunity for teaching, learning, and success in contributing to the mission” (NASA 2014). This paper outlines the history, development, implementation, and future plans for including undergraduate students and the public in the Psyche mission.

### Psyche Student Collaborations Program History

NASA has a decades-long legacy of planetary science educational and public outreach efforts (Table 1; see also agency-wide program summaries in e.g., Kaminski 2021; Smith et al. 2014) at the programmatic (e.g., Mars Public Engagement), flagship mission (e.g., Cassini), and principal investigator-led mission (e.g., Dawn, Lucy, MAVEN) levels. In developing the Psyche Student Collaborations program, the team was informed by many previous and ongoing efforts within specific NASA divisions and missions (e.g., Cassini Inspires, Send Your Name to Mars, JWST Art, Dawn Journal blogs) as well as agency-wide initiatives (e.g., Science Activation Program, Museum Alliance [now the NASA Museum and Informal Education Alliance], Solar System Ambassadors, NASA Solve, NASA Space Place, Night Sky Network, and NASA Citizen Science).

**Table 1:** Selection of NASA program exemplars

Program	Description	URL
Cassini Inspires	A campaign that invited the public to create and post their artistic creations inspired by NASA’s Cassini mission on social media with the tag #CassiniInspires.	https://solarsystem.nasa.gov/resources/17598/cassini-inspires/
Send Your Name to Mars	A campaign program that encourages space enthusiasts to submit their name to be etched onto a microchip that will be carried by a Mars rover.	https://mars.nasa.gov/participate/send-your-name/
JWST Art	An artistic program collaborating with interns and the public for the creation of art about the James Webb Space Telescope.	https://webb.nasa.gov/content/features/jwstArt/publicArt.html
Dawn Journal Blogs	A NASA blog that provided journal entry descriptions discussing Dawn mission milestones with the public.	https://solarsystem.nasa.gov/missions/dawn/mission/dawn-journal/
Science Activation	A cooperative network connecting NASA science experts, content, and experiences with community leaders.	https://science.nasa.gov/learners
Museum & Informal Edu. Alliance	An education program providing free NASA educational resources and services to educators and the general public.	https://informal.jpl.nasa.gov/museum/
Solar System Ambassadors	A public engagement program using nationwide volunteers to communicate the science and excitement of NASA’s missions and discoveries.	https://solarsystem.nasa.gov/solar-system-ambassadors/
NASA Solve	A website inviting the public to solve problems on NASA projects and participate in research and prize competitions.	https://www.nasa.gov/solve/
NASA Space Place	A website featuring games, articles, videos, and hands-on activities meant to educate children on space and Earth science.	https://spaceplace.nasa.gov/
Night Sky Network	A nationwide coalition of astronomy clubs sharing information about NASA missions and telescopic observations with the general public at museums, observatories, and classrooms.	https://nightsky.jpl.nasa.gov/
NASA Citizen Science	Scientific project collaborations between NASA scientists and public volunteers interested in participating in research.	https://science.nasa.gov/citizenscience

Through the Discovery proposal process, the program design was refined with feedback from NASA’s Science Mission Directorate, culminating in the present program, which leverages the enthusiasm and contributions of undergraduates nationwide to the benefit of the mission, the students and their institutions and communities, and the broader public. The Psyche Student Collaborations logic model (Appendix A) shows the alignment of the present program to the latest strategic goals outlined in the NASA Strategy for STEM (Science, Technology, Engineering, and Math) Engagement 2020-2023: “Create unique opportunities for a diverse set of students to contribute to NASA’s work in exploration and discovery”; “Build a diverse future STEM workforce by engaging students in authentic learning experiences with NASA’s people, content and facilities”; “Attract diverse groups of students to STEM through learning opportunities that spark interest and provide connections to NASA’s mission and work” (NASA 2020).

### The Discovery 2014 Announcement of Opportunity: The “Charge” for Student Collaborations

The Discovery 2014 Announcement of Opportunity (AO) reiterated that “among NASA’s strategic goals is to communicate the results of its efforts to the American public and to enhance the science and technical education of the next generation of Americans” (NASA 2014). Though an Education and Communication plan was not requested in the AO, proposers were invited to submit an optional Student Collaboration plan. The language in the AO suggested that an expected Student Collaboration opportunity could “take the form of an instrument development, an investigation of scientific questions, analysis and display of data, development of supporting hardware or software, or other aspects of the investigation”, likely something like a camera, requiring that the proposal “provide details of the development schedule of the SC [Student Collaboration], including decision points for determining SC readiness for flight” (NASA 2014). In Discovery 2014, optional proposals for Student Collaboration projects were expected to be aimed at university undergraduates only, and with a budget up to 1% of the proposal cost cap, which for that call for Phases A-D was $450M. Psyche proposed for the full amount spread over the full life of the mission, equaling slightly over $400,000 per year (the majority of the budget is dedicated to one full-time lead, supported by up to 6 student interns, with the remainder dedicated to supporting the program elements detailed below).

During the Step 1 proposal process, the Psyche mission proposal team proposed a cubesat as a Technology Demonstration Option with associated Student Collaboration opportunities. Upon updated NASA guidance indicating that cubesats would count against total mission risk, the Psyche mission proposal team removed the cubesat element from the proposal. The team instead outlined activities for Student Collaboration related to instrument development and science operation opportunities, as well as research projects. Upon selection in 2017, however, NASA challenged the mission team to produce a more ambitious Student Collaboration program than previously proposed. The mission team entered a few months of conceptualization and iteration to finalize the current program with NASA (see Appendix B for a description of additional projects proposed after selection but not included in the final program). The goal was to include a strong element of interdisciplinarity in the program components to reflect the reality of working in teams at NASA and other large, complex endeavors, rather than the single-discipline experiences undergraduate students tend to encounter in their coursework and degree programs (NASEM 2018).

### Psyche Student Collaborations Guiding Concepts

The guiding concepts for the Psyche Student Collaborations program reflect NASA’s strategic goals as well as the charter of Arizona State University (ASU), which states, “ASU is a comprehensive public research university, measured not by whom we exclude, but rather by whom we include and how they succeed” (ASU 2014). The program is designed to:

- Attract and train future instrument and mission leads, both science and engineering;
- Facilitate innovation by including people from fields outside science and engineering, including the arts and humanities;
- Offer a portfolio of programs that range in the extent of direct engagement with mission team members and the numbers of participants who can participate (e.g., from small numbers of internships working directly with team members to thousands able to take the asynchronous free online courses available worldwide);
- Extend through the life of the mission, starting in Phase B, and;
- Include ways for people at any level of education or nationality to participate through opportunities developed and facilitated by undergraduate science outreach interns.

Additionally, the mission team holds as a core value that there are space exploration-related jobs in all disciplines and that learning to value all disciplines within interdisciplinary teams is a key skill for exploration and for everyday life. These guiding concepts are addressed by four main program components and two cross-cutting efforts (evaluation; publication and dissemination).

### Psyche Student Collaborations Program Components and Timeline

Psyche Student Collaborations consists of four main programs, two of which are available solely to undergraduates enrolled at universities or community colleges in the United States and its territories (Psyche capstone projects and Psyche Inspired, outlined in Sect. 4) and two of which invite broader participation and leverage the talents and creativity of undergraduate student workers to help create content to engage the public and lifelong learners (Innovation Toolkit online courses and Psyche Outreach Interns and Docents, outlined in Sect. 6). These programs are managed by one full-time research faculty member at ASU with a team of six part-time ASU undergraduate student workers. The program benefits from voluntary in-kind contributions (such as STEM expertise, presentations, content review, social media amplification, etc.) from Psyche mission team members at NASA, JPL, mission partner institutions, and members of the ASU community and the School of Earth and Space Exploration.

- Psyche Capstone Projects (https://psyche.asu.edu/get-involved/capstone-projects/): Capstone courses (also called Senior Design) are culminating, project-based courses undertaken by university students in any major in the final (senior) year of university. Psyche develops mission-aligned capstone projects undertaken by teams of university capstone students nationwide in engineering, the sciences, the humanities, and the arts.
- Psyche Inspired (https://psyche.asu.edu/get-involved/psyche-inspired/): Psyche Inspired is a virtual “creatives-in-residence” program that brings undergraduate students from any discipline or major together to share the excitement, innovation, and scientific and engineering content of NASA’s Psyche mission with the public in new ways through artistic and creative works.
- Innovation Toolkit Online Courses (https://psyche.asu.edu/get-involved/innovation- toolkit/): A growing series of free, asynchronous online courses based on the real-world challenges and skills associated with the mission’s science, engineering, technology, and teamwork.
- Psyche Science Outreach Interns and Docents to Support Public Engagement (https://psyche.asu.edu/get-involved/): These student workers form the majority of the Psyche Student Collaborations team and support the program components and public engagement efforts while gaining valuable work experience.

**Fig. 1:**
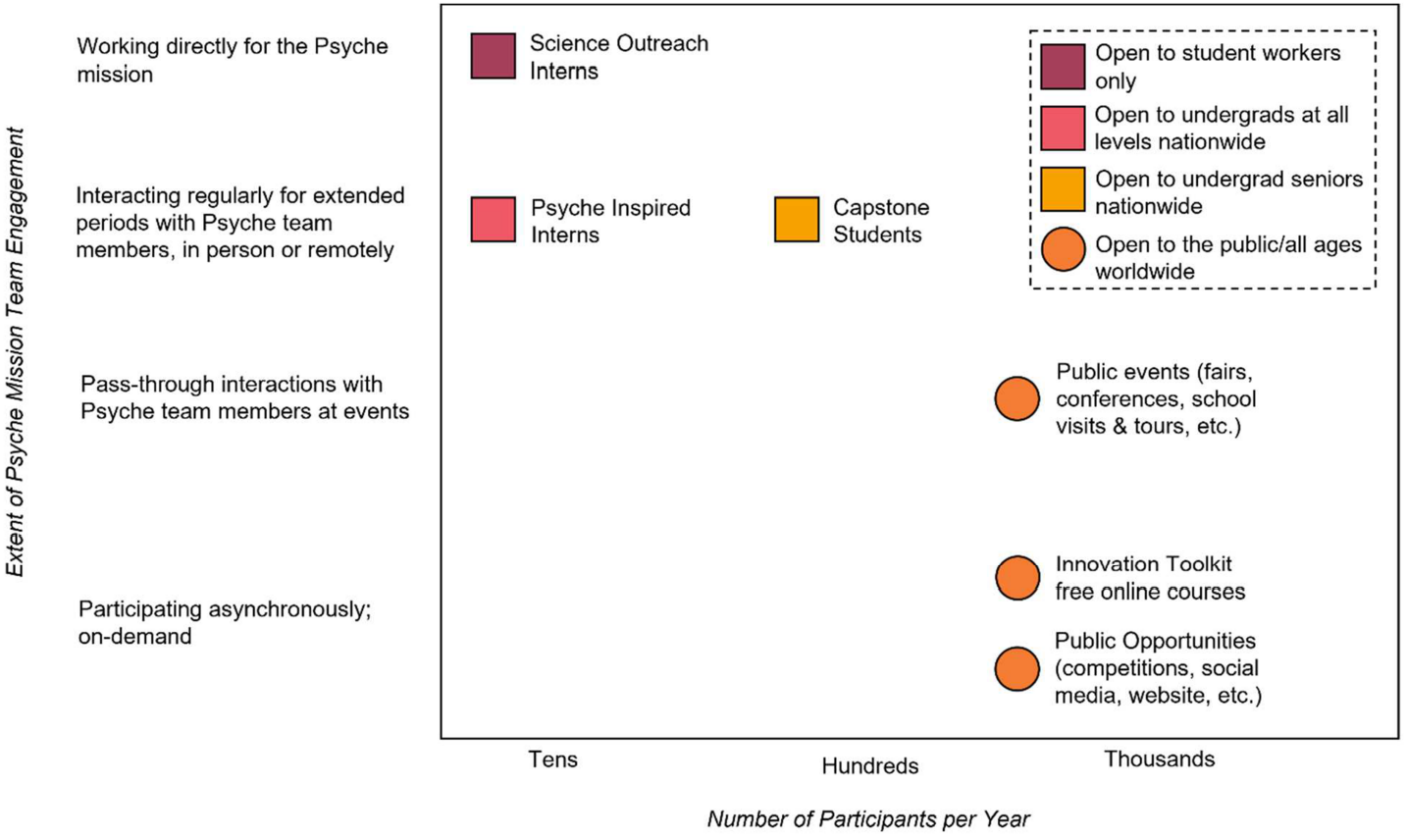
The portfolio of Psyche Student Collaborations programs balances intensity of direct engagement of mission team members with numbers of participants and mode of delivery (e.g., in person; remote but synchronous; remote but asynchronous). In this figure, programs indicated by a square are for undergraduate participants and programs indicated by a circle are for the general public of all ages

Additionally, working with Psyche public engagement, public affairs, and media relations team members at NASA, the Jet Propulsion Laboratory (JPL), and partner institutions, the program supports the development of outreach products and public engagement opportunities, including the mission website, social media, video products, and public events (outlined in Sect. 4). These offerings provide multiple entry points and present experiences of varying intensity and time commitment, accommodate differing numbers of participants, and offer in person and remote modes of delivery (Fig. 1).

These program components have strong support and extensive voluntary participation from mission team members at all levels and from many disciplines, including mission leadership, management, scientists, engineers, communications professionals, and student interns. The results are documented and disseminated through ongoing evaluation and publication.

The program components were piloted at ASU in the early part of the mission’s Phase B (2017-2018 academic year) and expanded nationally beginning with the 2018-2019 academic year. Program participation and reach continues to grow and active participation by students and the public is planned through Phase E of the mission. Phase F of the mission will focus on final documentation and publication.

### Mission Team Member Involvement

All members of the broader mission team (from NASA, JPL, and partner universities and institutions) are made aware of opportunities to contribute to Psyche Student Collaborations efforts. As with the programs for undergraduates, opportunities for mission team members to be involved cover a range of offerings that vary in terms of intensity and commitment so they may choose what works best for them given their interests, preferences, and available time. Activities with greater time commitment or intensity of engagement include providing scientific or technical advising for capstone projects, crafting capstone project briefs, reviewing Psyche Inspired applications, creating and reviewing content for Psyche online courses, and writing abstracts and journal articles (such as the present article). Activities requiring less intensity or time commitment include being guest speakers for capstone teams or Psyche Inspired, providing suggestions for social media content, giving public talks or participating in classroom visits, assisting in writing or reviewing Frequently Asked Questions (FAQs) or content for the website, testing new student products, or answering questions that come through the public contact form or social media. At any given time, about 10% of the Psyche team (along with non-Psyche team members from NASA, industry, universities, and the arts) is actively participating in one or more of these activities, ranging from 30 minutes to 50 hours per year per team member, depending on the activity. Additionally, dozens of Psyche capstone, Psyche Inspired, and science outreach intern and docent alumni stay involved through offering job-seeking advice to current students, supporting networking events, or advising on their projects’ next steps on an “on-call” basis.

## Undergraduate Participation

The design of the three main programs open specifically to undergraduate students (Psyche capstone projects, Psyche Inspired, and the Psyche outreach interns and docents) seeks to strike a balance between depth of engagement with mission personnel and the reach of the programs and ability to scale to include greater numbers of students.

### Cumulative Undergraduate Participation to Date

**Fig. 2:**
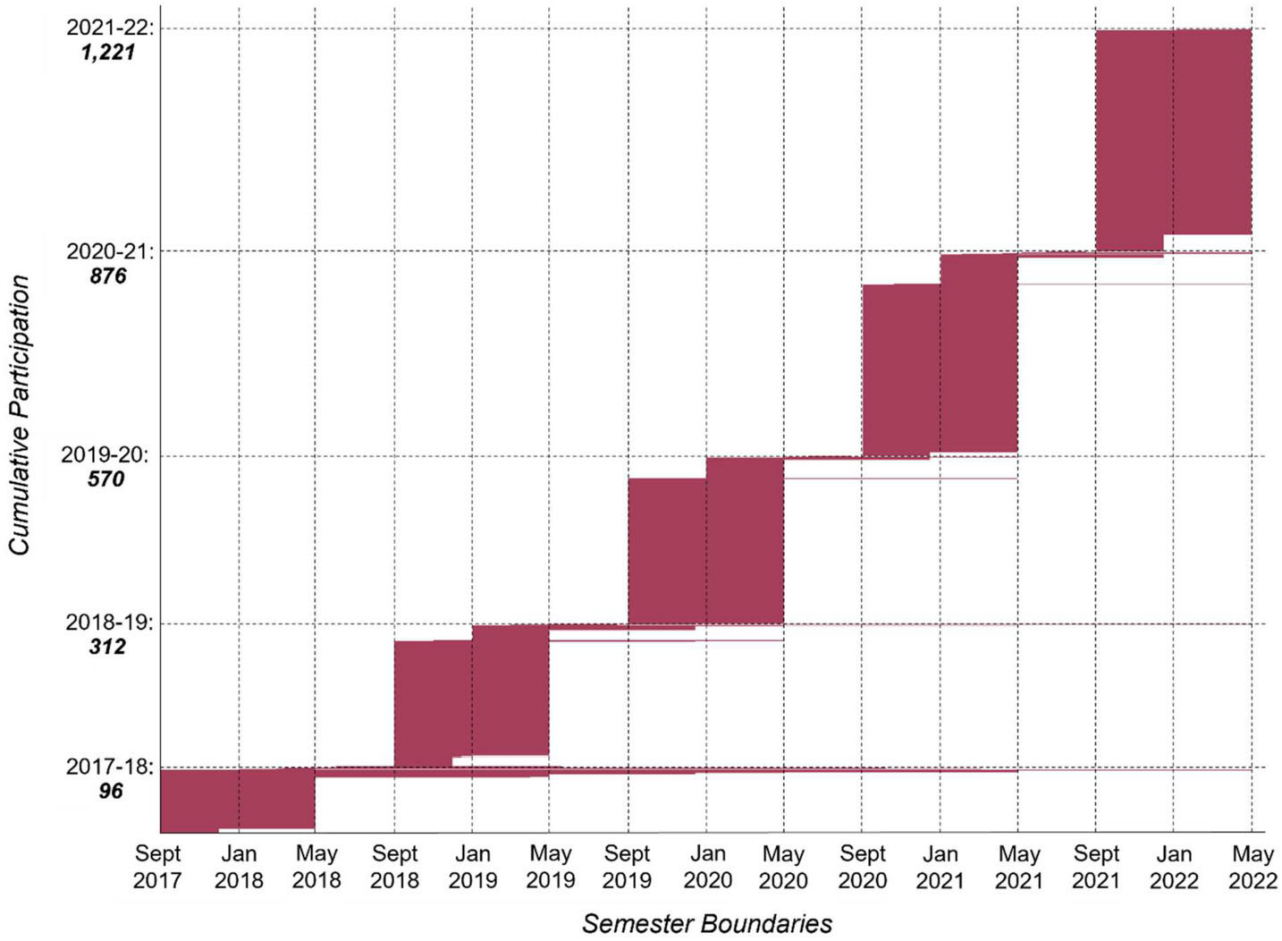
Cumulative undergraduate participation (n = 1,221) September 2017 - May 2022 (chart concept credit: R. Binzel). The bulk of the participants every year are part of the Psyche capstone program (n = 1,108) followed by Psyche Inspired (n = 75) and science outreach interns (n = 24) and docents (n = 14). Some participants take part in multiple programs or participate for multiple years (such as interns and docents) but they are only counted once horizontally

Over the course of five academic years (from fall 2017 to spring 2022), more than 1,200 undergraduates have participated in Psyche Student Collaborations through the Psyche Capstone program, Psyche Inspired, and as Psyche Outreach Interns and Docents. Figure 2 shows cumulative participation. The programs provide deep and extended engagement; at a minimum, students participate for one semester, but most participate for two semesters. Some students continue over time and some have participated in multiple programs (in this figure, if a student participates in multiple programs or continues over time, they are not “added” vertically but may be seen extending horizontally).

#### Mission Contact and Participant Effort

**Fig. 3:**
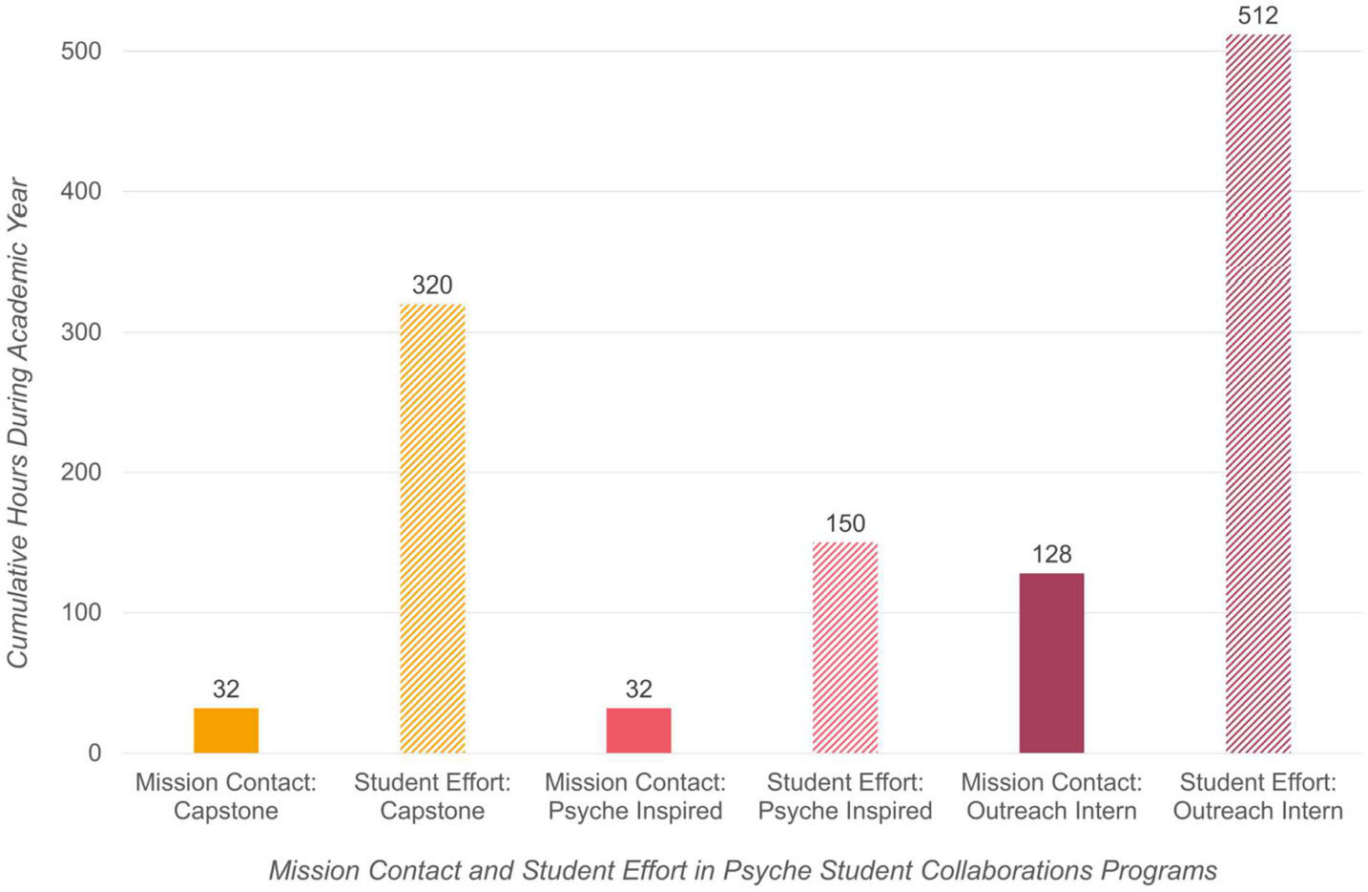
Estimated cumulative contact hours (with mission personnel) and effort hours (minimum time *each* participant is expected to work on their project [capstone and Psyche Inspired] or paid to work [outreach interns]) during the academic year quantify the substantial contribution that undergraduate participants make to the mission through Psyche Student Collaborations beyond the direct contact they have with mission personnel. For example, each Psyche Inspired intern has a minimum of 32 hours of contact with mission personnel and contributes a minimum of 150 hours of work on their project

The estimated minimum average contact hours and effort hours of each type of participant in the undergraduate programs is shown in Fig. 3. “Contact hours” in the figure refer to direct interaction between mission personnel and the participant, whether synchronous (such as in meetings) or asynchronous (emails, messaging). Capstone program participants meet in teams with the project sponsor (and other technical and scientific advisors as appropriate) 30 minutes biweekly, with the remaining contact hours with either the team or individual team members through communication on an online messaging platform or emails, feedback on deliverables, connecting them with resources, and attendance at mission presentations and question-and-answer sessions. Psyche Inspired participants meet as a group with program staff and special speakers one hour per week, with incidental communication individually through an online messaging platform as needed to support their progress. Each Science Outreach Intern has contact (individually or in groups) with mission personnel approximately an hour per day when they are working (an average of four days per week). “Effort hours” in the figure refer to expected individual participant effort or contribution related to the mission. Capstone program participants, who are receiving course credit, are each expected by their course faculty to each contribute ten hours per week to their project. To accomplish their creative works (from ideation to proposal to execution), each Psyche Inspired participant is expected to spend five hours per week on their projects at a minimum, though for some it may be much more, depending on the project scope and medium. Science Outreach Interns, who are paid by the mission, each work an average of 16 hours per week (beyond contact time with mission personnel, which is approximately 4 hours per week).

The undergraduate programs, initially piloted at ASU during the 2017-2018 academic year, expanded nationally at the start of the 2018-2019 academic year and have involved undergraduate participants at 50 universities or community colleges through the 2021-2022 academic year (see Appendix C, Table 4) in 25 states with students majoring in dozens of fields and disciplines across the arts, humanities, sciences, and engineering (see Appendix D, Table 5). The programs continue to expand and diversify in terms of geographic location (of students and institutions), institution types (university, college, community college), and participating major or discipline.

#### Alumni Engagement

The hundreds of undergraduates that work with Psyche Student Collaborations each year will culminate in thousands of former participants by the end of the mission. As part of their final capstone project, in fall semester 2017 a Public Relations Psyche capstone team recommended the creation of an alumni program and suggested that the classes be designated with names, rather than years, since not all participants are seniors when engaging with Psyche Student Collaborations (for example, as science interns and docents or in Psyche Inspired). The naming convention adopted was a list of metals that may be found at Psyche (Elkins-Tanton et al. 2020, 2022), ordered by their number on the periodic (Appendix E, Table 6). The Psyche Student Collaborations team maintains private LinkedIn and Slack groups to connect with alumni and post internship and employment opportunities in all disciplines, highlight upcoming events, and share mission updates. A recent scan of current status of the alumni connected with the Psyche LinkedIn alumni group include 38 that are working for government agencies or non-profits, 53 that are still students (either finishing undergraduate degrees or in graduate school), and 380 that are working in industry. A periodic email newsletter is sent containing similar information. Alumni are able to reach out to Psyche Student Collaborations team members to request references for jobs, letters of recommendation for graduate school, and advice on postgraduate studies and careers.

Alumni have also proven to be a valuable source of advice and contacts for current participants. For example, Psyche capstone alumni were contacted in August 2020 to share advice (anonymously) they would like to give to incoming capstone students. Their responses were aggregated and became part of a presentation, “Use Capstone to Your Advantage,” presented to ASU’s Computer Science (CS) and Computer Systems Engineering (CSE) capstone students at the start of the 2020 fall semester. Themes of their advice included: Focus on teamwork and communication; make sure to document your work; network and build long-term relationships (as one noted, “Your teammates will be your long-term connections and possibly your references in job applications so try to foster a closely knit team dynamic”); be practical; and stretch yourself and have fun. Many alumni have also been willing to connect with current students to discuss their workplace, share tips on the job search, and even advise on continuing projects they worked on previously. This has been especially useful for students participating in the Psyche capstone program since most are graduating at the end of the program.

### Psyche Capstone

Capstone courses (also known as Senior Design) are culminating, project-based courses undertaken by university students in any major in the final (senior) year of university. Although there is some variation in terms of logistics and implementation of different capstone or senior design programs (see e.g., Howe et al. 2017), a common strength of capstone courses is that they involve students in applying their knowledge and skills to real-world, team-based projects and “[offer] closure and a focus for the sense of achievement that comes with completion” (Lee and Loton 2015). With over 70% of colleges and universities offering capstone courses, Hauhart and Grahe (2015) estimate that approximately a million U.S. college students participate in capstone projects annually.

Although specific logistics and structures may vary by course, department, and university, in general they involve teams of students, course faculty, project sponsors or mentors, and the projects themselves (see Howe et al. 2017). Students form or are assigned by their professor to teams of an average of three to five students (or more, depending on the course or project need). The team selects or is assigned to a project based on a project brief, which is often written by the project sponsor (most commonly a company or faculty member), though sometimes formulated by the students themselves. The project brief outlines the motivation for the project, the desired outcome, and other relevant background information. In undertaking the project, the student team meets with the sponsor regularly, follows the schedule of deliverables required by the capstone course, and engages in independent and group work in pursuit of the project outcome. The ubiquity and diversity of capstone and senior design courses at colleges and universities around the country offers an accessible and sustainable way for missions to engage with undergraduates in small groups or at scale.

#### Program History and Structure

The Psyche capstone program started in the 2017-2018 academic year. To facilitate broad adoption, the program focuses on developing standalone capstone projects that may be undertaken by students in any capstone or senior design course (or across capstone courses in multiple disciplines) with relevant interests and abilities. Psyche projects range from the practical (a digital publication repository and archive) to the exploratory (automated meteorite image analysis) to the futuristic (robotic explorers designed to accommodate all potential hypothesized surface characteristics of the asteroid for as-yet unplanned future exploration). Some projects are designed to inform and enhance the Student Collaborations program directly. Examples include the creation of an online interdisciplinary capstone “marketplace” to match students with projects across disciplines, evaluation of the Psyche Inspired program, and an analysis of ways to optimize the capstone program itself to enable scalability. Other projects may inform the decision making and development of future mission public engagement opportunities, such as communications campaigns and comparative analyses of previous mission opportunities (discussed in Sect. 5). The process of capstone project formulation is discussed in Sect. 2.2.3.

Given the large variation across institutions in the structure, content, and administration of capstone courses (Howe et al. 2017), Psyche projects are intentionally open and flexible to being modified and adapted to accommodate the needs of participating faculty and courses. As an example, during the 2020-2021 academic year three teams from different departments and universities each worked on the project brief “Planetary Geologic Mapping of a Hypothesized Surface,” which was proposed by a Psyche Co-Investigator. Each team chose to approach the project in a different way based on their experience, career interests, and requirements of their capstone courses. One, an interdisciplinary team made up of astronomy, geology, and engineering systems design students, focused on forecasting and implementing a feasible geologic map and developing methods for identifying interesting as well as safe landing sites for hypothesized surface explorers. A computer science team from a capstone course that is open to research-style projects chose to try to implement code capable of automatically detecting surface features. A computer science team from a different university whose course requires product development focused on creating a webpage capable of displaying ArcGIS maps of planetary surfaces (including the artist’s rendition of the Psyche surface) and explaining core concepts of geologic mapping for users.

Being flexible to these types of variables has been a key element in the development of project briefs from the start of the program, which was piloted during the academic year 2017-2018. The pilot year involved 10 teams of students at ASU working on four project briefs (with an additional two teams working on capstone-style projects but in a non-capstone course), including five interdisciplinary teams made up of computer science, graphic design, and engineering management students who worked to create a mobile app for the mission. From the start in 2017 through the 2021-2022 academic year, the program has engaged 19 university systems (see Appendix C) and worked with over 1,100 capstone students from majors in various fields of engineering, science, communications, and the arts (see Appendix D). At present capacity, the program engages approximately 300 capstone students in over 60 capstone teams each academic year.

Since exploration endeavors such as the Psyche mission, or any other complex, real-world problem, require the skills of a wide range of disciplines working together, the mission strongly encourages interdisciplinary capstone teams where possible. As few universities offer interdisciplinary or multidisciplinary capstone courses, the program works with capstone partners to offer interdisciplinary projects that can incorporate and accommodate students enrolled in separate single-discipline capstone courses. Through these projects, students from different disciplines work together on the project as an integrated interdisciplinary team while still meeting the deliverables of their individual capstone courses. In some cases, students from multiple universities work together as a team, providing an opportunity to prepare them to engage with a diversity of disciplines and collaborate in remote teams in the workplace, as is typical of large-scale projects like space missions. Engineering students have seldom worked on project teams with graphic design students, for example, and graphic design have seldom worked on projects guided by project managers, but such scenarios are common in the workplace and this experience can help provide them with important preparation. Additionally, through such capstone projects, universities may gain access to non-local, specialized technical mentors and to disciplines not offered at their institutions.

Aligned with the diversity of modern universities and the modern workplace, the mission has formed seven different team composition types to date (Table 2). ASU offers many online degree programs (https://asuonline.asu.edu/online-degree-programs/) and to-date the Psyche capstone program has worked with online students in engineering management (who often join other hybrid teams as project managers), software engineering, and electrical engineering. As online degree programs with online capstone course sequences become more common (both at ASU and at other partner universities), there may be opportunities in the future for online students from multiple disciplines and from multiple universities to team together in capstone.

**Table 2:** Capstone team compositions

	All On-Campus Students	All Online Students	On-Campus & Online Students
Single University, Single Discipline	•	•	•
Single University, Multiple Disciplines	•		•
Multiple Universities, Multiple Disciplines	•		•

Regardless of the type of team, the students receive some measure of structure from their capstone course itself. The Psyche capstone program also provides all participating students with a secondary, overarching structure to facilitate communication, professional development, and exposure to the mission. Key elements include onboarding (an introductory presentation about the mission, the Psyche team guidelines, team members and contact information; an Intellectual Property [IP] agreement to be signed; and other administrative tasks), a range of program communication and information options (messaging platforms [such as Slack, Ryver, and Basecamp], a shared Google drive, bi-weekly meetings with the program lead and relevant subject matter experts), and resources (mission speakers, optional one-on-one meetings with the program lead, technical support, training opportunities, relevant publications, and an alumni program). This program-level structure allows the mission to work with many universities and styles of capstone and senior design courses in a way that is sustainable in terms of staffing and resources and could be passed on to other missions or university entities interested in replicating or continuing the model following the conclusion of the Psyche mission.

#### Leveraging University Partnerships

Arizona State University (ASU), with more than 100,000 students spanning multiple campuses as well as a large online program, provided the opportunity to expand rapidly even in the pilot year of the program. Though there was no centralized office for capstone courses university-wide, a science outreach intern created an internal database of potential faculty contacts by searching the digital course catalog for senior-level courses with keywords such as “capstone”, “senior design”, or “project-based”, as well as reviewing online undergraduate major maps. The Student Collaborations team approached faculty to arrange individual introductory meetings and then hosted a kick-off meeting in August 2017 for capstone faculty from multiple disciplines and ASU campuses to meet. The outcome was a pilot year involving 12 capstone (or capstone-style) teams with members from computer science, computer systems engineering, communications, graphic design, and engineering management, and the creation of an ASU-wide capstone email listserv with over 100 members. As planned, the following year the program expanded nationally, with partners solicited via conference participation (e.g., 2018 Capstone Design Conference), word-of-mouth (within ASU and beyond), social media posts directing interested participants (whether faculty or students) to submit their details via the online interest form, and, importantly, direct referrals from mission team members. Referrals from mission team members may be in the form of a direct contact at their alma mater, a colleague working at a university, or a previous institutional partnership.

As an example, NASA’s Glenn Research Center (GRC) is located in Cleveland, OH and has a long history of providing capstone projects and mentorship to student teams at Cleveland State University (CSU) in the Washkewicz College of Engineering. Through their prior connection, Psyche team members at GRC offered an introduction to the CSU capstone coordinator, as well as applied their subject matter expertise in solar electric propulsion (SEP) to the development of capstone project briefs and subsequent technical and scientific mentoring (both of CSU teams and others, see Sect. 2.2.3). The direct referral from GRC served as a “stamp of approval” for the emergent Psyche capstone program, providing a strong foundation from which the partnership with CSU has grown and a model for other direct referrals that may be possible in the future. Through the 2021-2022 academic year, the Psyche capstone program has worked with CSU capstone teams each year since national expansion, including with teams focused on GRC-generated projects related to SEP (such as Xenon Flow Controller; Hall Thruster Database; and Hall Thruster Visualization), with teams receiving technical and scientific mentorship from GRC mission team members.

#### Project Formulation and Mentoring

Capstone project briefs are solicited from mission team members annually and result in 15-25 distinct project briefs each year. The criteria for projects is that they are relevant to the mission but not in the critical path—ideally projects that the mission team member thinks would be a good idea to try or would be nice to have completed but has not had the time or resources to pursue themselves. Projects may be intended to be completed in a semester, an academic year, or across multiple academic years by multiple teams. As appropriate, projects may be modified to accommodate student interests, skills, or previous coursework, and new projects or extensions to previous projects may be added. Mission team members submitting projects are not required to provide scientific or technical mentoring of the projects but are welcome to participate at the level they choose, and this varies by team member and project. Below are examples from mission team members involved in capstone, which illustrate the range of approaches taken in formulating projects and providing scientific or technical guidance and mentoring. Formulation of Psyche capstone projects generally follows four approaches (see summary in Table 3): 1) a specific, self-contained challenge faced during mission development; 2) the opportunity to re-think or refine existing solutions through the fresh perspectives and skills of students in interdisciplinary teams; 3) a standalone project inspired by, or of use to, the Psyche team; and, 4) future-looking projects that push students, as well as science and technical mentors, to explore solutions to challenging “what if” scenarios.

**Table 3:** Summary of Psyche capstone project formulation approaches

Approach	Description	Example Project(s)
1) Self-contained challenge	A general space mission development challenge is isolated for student teams to explore areas of engineering that may have been unexplored or underexplored during spacecraft development and may prove fruitful in future applications	• Xenon Flow Controller
2) Harness fresh perspectives for existing challenges	Fresh perspectives & new skills of students are used to further a field of inquiry faced as part of the Psyche development that may not have been fully solved or that resulted in the opening of additional avenues for research.	• Machine Learning Analysis of HET Facility Effects • Solar Wind Propagation • Meteorite Image Analysis
2) Mission team standalone projects	Standalone projects inspired by the Psyche mission team. Deliverables provide tools that can be leveraged by the mentor and the mission to further educational and public outreach goals while also providing the student teams the opportunity to directly contribute to an active NASA mission.	• Hall Thruster Visualization and Animation Project • 3-D Asteroid Viewer • Science Activity Planner
4) Future-looking projects	Future-looking projects that push students, as well as science and technical mentors, to create solutions to challenging “what if” scenarios.	• Hypothesized Surface Exploration (Landers, Robotics, Sampling, etc.)

One project formulation approach is to isolate a self-contained challenge faced during general space mission development and present it to the students. The Xenon Flow Controller project from the 2018-2019 academic year is an example of this approach. In this project, student teams from Cleveland State University (CSU), Arizona State University (ASU), and Florida A&M University-Florida State University (FAMU-FSU) were asked to design a system to control the flow of xenon propellant to the electric propulsion system used by the spacecraft and described in detail in Snyder et al. (2020a). The students were given similar requirements to those imposed on the system actually developed for the spacecraft and provided an opportunity to apply their classroom knowledge to replicate the real-world engineering processes that lead to closure of that design challenge on the actual spacecraft. From the mentor’s perspective, this approach provides the unique opportunity to leverage the creativity of student teams to explore areas of the engineering trade space that may have been unexplored or underexplored during spacecraft development but may prove fruitful for further development or future applications. Given that a known engineering solution exists for the projects developed in this project formulation approach, the challenge for the mentor is to avoid reducing the project to a rederivation of that solution. Instead, the mentor is challenged to support and guide the student teams towards a technically viable solution without stifling creative alternative solutions.

A second formulation approach is to harness the fresh perspectives or new skills of student teams from different technical disciplines in order to further a field of inquiry faced by the mission that may not have been fully solved or that resulted in the opening of additional avenues for research. An example of this approach is the Machine Learning Analysis of Hall Thruster Facility Effects Data project from the 2019-2020 academic year. One of the challenges facing the spacecraft is the difficulty in predicting on-orbit performance of the spacecraft’s electric propulsion system due to the known sensitivity of these devices to their operational environment and the inability to perfectly replicate orbital conditions on the ground (Snyder et al. 2020b). For the mission, detailed performance measurements were taken in the lab pre-launch and used to create a model for approximating on-orbit performance. In this project, computer science student teams were presented with the same data and asked to apply advanced machine learning techniques in order to determine if these more advanced techniques could improve the performance predictions and uncover previously unexplored sensitivities and trends. For the students, this approach provides an opportunity to apply their classroom knowledge to a real-world problem while, for the mentor, it provides an opportunity to explore new approaches and methodologies to further their technical inquiries. Here the mentor is challenged with distilling their central research problem so that it is understandable to student teams outside their core discipline and navigating that difference in disciplines in order to achieve a mutually beneficial outcome.

The Solar Wind Machine Learning Propagation Model capstone project is another example of such an approach. This project is directly related to the magnetometry investigation, which is tasked with determining whether the asteroid is magnetized and to what extent. Since the asteroid’s field would be distorted by the solar wind flow around it, knowledge of the solar wind conditions at the spacecraft can assist in interpreting the measurements. Since the flight system does not carry instrumentation that directly measures the wind, the magnetometry investigation will use concurrent observations of the solar wind measured by other spacecraft near Earth to estimate the wind condition at the spacecraft. However, accurate predictions can only be achieved for certain alignments of the spacecraft, Earth, and the Sun.

The solar wind propagation model capstone project explored the possibility that a machine-learning based model can out-perform existing methods, designed as an exploratory project that complements efforts within the Magnetometry Investigation team to build a non-machine learning based model. In the first iteration of this project brief (during the 2019-2020 academic year), the students were undergraduates attending ASU, with technical advising from Magnetometry Investigation members from Massachusetts Institute of Technology (MIT). Two teams worked in parallel on the project. For the students, this project offered the opportunity to learn about a real-world problem facing the science team: how to remove uncertainty from the observations and make a more accurate interpretation of the data. Furthermore, this problem is open-ended and stands at the forefront of current research efforts. As such, it allowed the students to carve their own path and explore different methods. The student teams also self-organized and divided the project into subtasks between different participants. Some tasks were carried out by several students independently, which provided a means for comparing different machine learning approaches.

For the mentors, this project provided the advantage of taking on a wholly different approach to a problem already in the works within the science team, without diverting team resources to exploring this avenue. This was particularly suitable to a machine-learning based project: the field of machine learning is a growing field and new approaches and software platforms are being constantly introduced. At the same time, the scientific return from machine learning models, specifically in fields like space physics, is still uncertain. It is therefore more suitable to use such methods for exploratory projects, rather than putting them on the critical path of mission design and planning. The outcome of the first iteration of the project was that several students succeeded in producing software tools for processing data from existing missions and designing preliminary predictive models. These were later used to inform the magnetometry investigation team in carrying on further projects. Lessons learned were also implemented in the second iteration of this capstone project during a subsequent academic year.

Challenges encountered during this project will help to more clearly define and manage future projects. For example, while the project was mostly aimed at computer science students familiar with machine learning, it still required students to develop an understanding of what the quantities in their machine learning models represent in the real world, so they could make meaningful inferences and be able to test the model. To achieve this, students were given written materials and short “crash course” presentations by the scientific advisor. However, retention and integration of such information by students from a different discipline is challenging. Another hurdle to student performance was clear and well-defined instructions. While mission scientists are accustomed to exploring open-ended questions with no “right answer”, undergraduate students are not often required to engage in such activities. However, while a more narrowly defined project can produce better performance from the students, the open-ended nature of the “real world” capstone project has the advantage of exposing the students to the challenges and frustrations of real discovery work, which can be a unique and inspiring experience.

Another project was formulated from an underappreciated challenge in planetary science missions–ensuring a robust set of data for comparison to mission results. The Meteorite Imaging Project addressed a fundamental weakness in current knowledge of the abundance of sulfur, phosphorus, and silica-bearing minerals within iron meteorites. Although the bulk of Psyche’s material was expected to be metal per the mission team’s pre-flight assessment, the light elements are important constituents of the core of the Earth and other terrestrial planets and are known from iron meteorites. The existing methods for quantifying these elements’ abundance in large slabs of iron meteorites is, however, tedious and underutilized.

The challenge put forth to the capstone teams was to harness the power of imaging, image processing, and feature recognition, with only initial user training of the system, to determine the abundances of these light elements in all available large slabs of iron meteorites in the collections of Arizona State University and the Smithsonian’s National Museum of Natural History. Over multiple years, teams have combined expertise from students in project management, engineering, photography, image analyses, and automated image processing. Faced with real world challenges, including the need to provide uniform illumination and viewing geometry, the ability to distinguish different minerals that contain these light elements, challenges in edge detection of these phases, and automated processing, the teams have developed a wide range of solutions to date, both from the engineering perspective and software development.

Teams over five academic years have benefited from this real-world experience and made, most notably, significant advances in the systems needed for image capture and software development. A side benefit is the growing relationship between the mentors and the institutions involved, including a number of virtual and in person consultations with experts in meteoritics and planetary science to whom a largely engineering-focused group might not otherwise be exposed. Though the project was put on hold due the pandemic, but continued starting with the 2021-2022 academic year.

A third approach is to present students with a standalone project inspired by, or of use to, the Psyche mission team specifically. The Hall Thruster Visualization and Animation Project from the 2019-2020 academic year is an example of this approach. In this project, computer science capstone teams were challenged to create an entertaining and interactive animation to educate a general audience on the electric propulsion system used on the spacecraft. The final project deliverables provided tools that could be leveraged by the mentor and the mission to further their educational and public outreach goals while simultaneously providing the student teams the unique opportunity to directly contribute to an active NASA mission.

Another example is a need identified by Psyche team members (and of use to researchers and the general public) to have an accessible, quick-glance way to determine what sorts of data are available for a given target of interest on a planetary object. A seemingly straightforward solution to this problem is a graphical presentation of the target (in this case, eventually the Psyche asteroid) with easy-to-find information on what data have been taken for each point on that target. There are some detailed and powerful tools to do this, in particular those developed by NASA’s Solar System Treks project (https://trek.nasa.gov) and JMARS (https://jmars.mars.asu.edu/). However, a use case exists for a less feature-rich tool to quickly identify the data sets acquired to date. The Psyche capstone 3-D Asteroid Viewer project was proposed with the aim to present to the user, through a web browser, a simple graphic of a target in the form of a globe which can be rotated on all axes, zoomed in and out quickly, etc. The graphic is normally presented in shaded relief. The user is able to zoom into an area of interest and query the tool to see what data have been acquired. This is provided in the form of outlines of data takes (e.g., image outlines) on the globe or the centers of such data takes. By pointing and clicking on a data take location, a list of what data are in the Planetary Data System becomes available. (These functions are similar to what was once provided by the browser-based Google Mars website prior to it being incorporated into the Google Earth computer program [see e.g., Google Earth Blog 2009; NASA 2009) As a recurring project since the 2018-2019 academic year, teams of students at several universities have been designing and developing this tool, learning to use specialized NASA products such as the Planetary Data System (https://pds.nasa.gov/) and NASA’s SPICE, “an information system… to assist NASA scientists in planning and interpreting scientific observations from space-borne instruments” (https://naif.jpl.nasa.gov/naif/), strengthening and building upon each other’s work each year. The Psyche team mentor at JPL (and other team members as appropriate) attends monthly or bimonthly videoconference meetings during the academic year. At each meeting, the tool is demonstrated online to the mentor, using a target with existing datasets from flyby or orbital missions (e.g., the Dawn mission at Vesta). Each step in the design and development process is thereby made clear and the mentor shares in the excitement of the project with the students. In addition, the mentor is able to provide advice on how to improve current characteristics of the tool and suggest future modifications or additional features prior to sharing the tool with the Psyche team or the public.

The Activity Planner capstone project, pursued by capstone teams at two different institutions (ASU and Penn State Behrend) in 2020-2021, is an example of a capstone project with significant positive impact to the mission. This project idea was inspired by the nature of the Psyche operations concept, where reference plans are generated during the Integrated Sequence Build process before the start of orbit operations and where activity absolute start times will change due to geometric epoch updates late in the uplink process. These concepts together mean that quickly understanding distinct classes of differences between the approved reference plans and the current baseline is essential to having confidence while approving the uplink products. Though not in the critical path for operations, the desire for a plan “diff” (difference) software tool on Psyche was a lesson learned from NASA’s Dawn mission. In addition to operations, the tool will be useful in development as well since many notional or preliminary baseline plans are already being created and understanding differences between them can speed analysis and validation.

Both teams designed tools that essentially consisted of two parts: one part which evaluates changes to activity plans and one part which displays those differences. The first part analyzes activity plan JSON files and determines which and how many activities were added or removed from one plan to another, as well as which activities have modified start times, durations, or parameters. Because activities can be timed relative to geometric epochs, the tools need to be able to distinguish between activity start times that have shifted due to a change in geometric epoch timing and activity start times that have shifted due to manual changes. The second part of the programs display the results of this analysis in an interactive format. Users can see timelines of activity plans side by side, see differences between the plans highlighted by color, and hover over activities in the timeline to display tooltips with more detail.

Both capstone teams that worked on the Activity Planner project produced tools that the Psyche operations team could use as-is, although there will be ongoing work at JPL to take the best ideas from both tools and create one final version in time for use in Operational Readiness Tests. The availability of this tool is such a large quality-of-life increase that current Psyche MPST and SOST members are excited to use it as soon as it is available to them. The project was designed to have positive outcomes for the students as well. For many, this was their first experience with a medium-scale software project that was less structured than a typical class project, so they got experience talking to customers and interpreting their responses to form use cases, as well as making wide-ranging decisions about implementation to best meet them. They were also coached by JPL engineers about best practices like unit testing, git flow, encapsulation, and more. Technology-wise, the teams gained experience with PyQT, dash, plotly, numpy/pandas, and JPL open-source activity- and time-related libraries.

A fourth approach is to formulate future-looking projects that push students, as well as science and technical mentors, to create solutions to challenging “what if” scenarios. For Psyche, these projects have centered on teams proposing, designing, simulating and/or prototyping solutions for mapping, landing, exploring, and sampling the range of hypothesized surfaces of Psyche. These projects are similar to the types of exploratory projects undertaken by researchers and mission formulation teams at NASA centers and universities and funded by programs like NASA Innovative Advanced Concepts (e.g., https://www.nasa.gov/directorates/spacetech/niac/2018_Phase_I_Phase_II/Shapeshifters_from_Science_Fiction_to_Science_Fact/).

#### Capstone Products

**Fig. 4:**
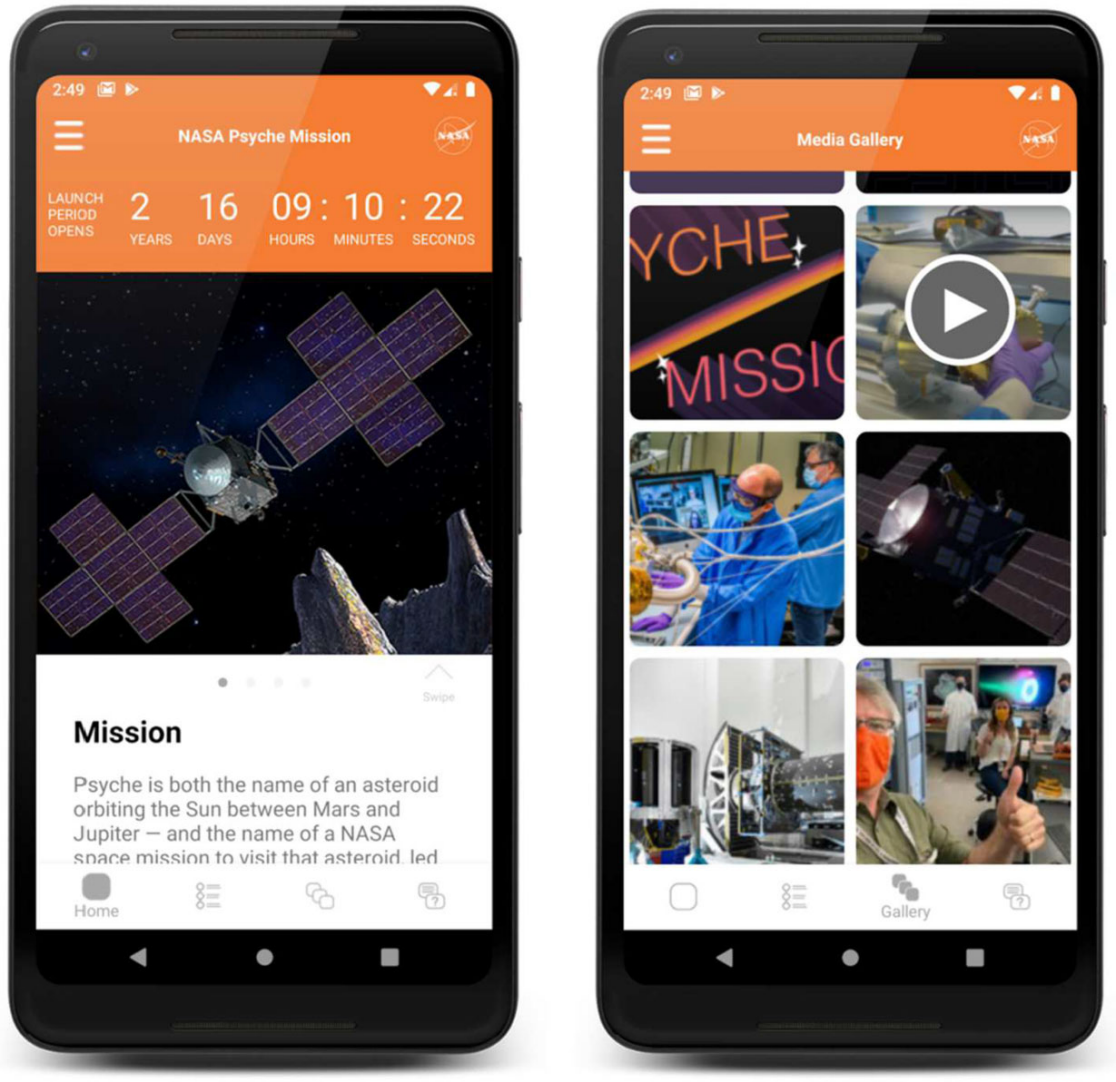
Capstone team-built mobile app

Capstone courses in all disciplines are structured so that the end of the course (if one semester) or course sequence (if two semesters) results in a final product from each team that is shared with the capstone project sponsor and showcased to their school and the public through a culminating event, presentation, or web-based summary. The course faculty structure the interim deliverables and assignments to help the teams reach this conclusion. Each team provides the sponsor with a final report and, if appropriate to their project, a final deliverable such as software, a prototype, or implementation plan. In the case of some Psyche projects that are especially challenging and long-term, requiring multiple years of teams to complete, the final deliverable includes handover materials (such as a github repository, equipment, instructions, recommendations, etc.) to guide a subsequent team. As projects are finalized, they are made available to the project advisors (such as with the Activity Planner) or shared with the public (as in the case of a public mobile app: https://psyche.asu.edu/mobile-app; Fig. 4). Regardless of the destination of the final product, each team has a dedicated page on the Psyche capstone program page with a URL that they may include on their resume to document their participation.

Beyond products from the capstone students themselves, the Psyche capstone program has resulted in presentations and posters at the *Capstone Design Conference* and the *Undergraduate Research Programs* conference, the *Council on Undergraduate Research* conference, as well as an article in the *International Journal of Engineering Education*. Additional dissemination and professional development efforts take place through a summer capstone faculty journal club organized by the Psyche capstone program team and a workshop on interdisciplinary, inter-university capstone teaming as part of the *Capstone Design Conference* virtual offerings in summer 2021. It is also hoped that the Psyche capstone program can provide a feasible model for other PI-lead missions to consider that are developing undergraduate-focused Student Collaborations plans.

#### Feedback and Program Improvement

The individual capstone courses and instructors conduct student- and team-level assessment, including soliciting capstone project sponsor feedback, and assign grades as directed by their department and institution. Additionally, the Psyche capstone program lead and the capstone student manager conduct an IRB-approved internal program-level evaluation using an adapted version of empowerment evaluation (Fetterman 2001) focused on stakeholder (program staff and participant) engagement. It is conducted electronically (via survey) and spread across the full academic year, in which students participate in “(a) developing a mission, vision, or unifying purpose [for their year of the program]; (b) taking stock or determining where the program stands, including strengths and weaknesses; and (c) planning for the future by establishing goals and [determining] strategies to accomplish program goals and objectives” (Fetterman 2001). Used with a previous NASA student program with distributed participants (Fetterman and Bowman 2002), the modified, survey-based empowerment evaluation process, repeated annually, provides an efficient way to engage participants in identifying the salient elements of the program, guiding iterative corrections and enabling continuous program improvement, and informing future adaptations as the needs of participants and other stakeholders change over the lifetime of the program (see discussion of the Psyche capstone evaluation in Bowman et al. 2019 and Talamante et al. 2022).

### Psyche Inspired

Space exploration is often thought to be dominated by engineers, scientists, and the imaginations of science fiction writers. But as in any large human endeavor, there is need and room for many kinds of expertise, including artists and other creators, as NASA has long recognized through groups and projects like The Studio at JPL (https://www.jpl.nasa.gov/thestudio), the Mars as Art exhibits (http://mars.nasa.gov/multimedia/marsasart/), the JWST Art campaign (https://www.jwst.nasa.gov/content/features/jwstArt/index.html), and many others). Psyche Inspired addresses this by bringing undergraduate students together, virtually, to spend an academic year as “creatives-in-residence” to share the excitement, innovation, and scientific and engineering content of the mission with the public through artistic and creative works. The Psyche asteroid, the mission’s destination, remains unique and mysterious, both because it will be the first metal-rich world ever explored and because it is not yet known what it looks like. Through a telescope, it looks like a bright spot; with radar, scientists have learned about its approximate shape and size, but not its appearance. This provides a special opportunity for creative exploration and for inspiring the public (and the mission team, e.g., Appendix F) and encouraging people worldwide to dream, innovate, and explore in their own lives.

#### Program History and Structure

Each year the interns are selected through an online application process that includes submission of sample works and written responses to four questions (example application details available at https://psyche.asu.edu/get-involved/psyche-inspired/psyche-inspired-application-2022/). The application is advertised as widely as possible through direct emails and email newsletters (e.g., NASA’s Museum & Informal Education Alliance email newsletter; NASA STEM Express), listservs, social media (both the mission’s social media accounts and those of collaborators and other NASA accounts), and national organizations that reach undergraduates at universities and community colleges. The program selects up to 16 students annually (approximately 10-30% of applicants, depending on the application cycle), in honor of Psyche’s place as the 16th asteroid discovered. The selected interns are supplied with a stipend to support the creation of 4 (or more if they wish) original creative works to excite and educate the public about Psyche. In addition to weekly virtual meetings, interns create connections with speakers brought in from various disciplines and roles related to the mission, partner institutions, or professional artists and other creators.

Psyche mission team members support the Psyche Inspired program by volunteering their time to review the submitted sample works and accompanying interpretive text during the application period through a streamlined process enabled by a custom web application developed by Psyche software engineering interns. Approximately 10 Psyche team members from science, engineering, management, and public engagement participate in this phase each year. Written responses to the application questions are reviewed and scored by the Psyche Inspired management team based at ASU (the Student Collaborations lead plus 2-4 interns, depending on the year), who review and score each application separately, following the selection process guidelines that are outlined for applicants in the application (see Appendix G).

The Psyche Inspired program was piloted during the 2017-2018 academic year with 13 competitively selected interns from ASU. This inaugural group was referred to as the Titanium Class, as part of the naming convention of the Psyche Alumni program (see Sect. 2.1.2). Since all students in the pilot program were on-campus ASU students, they met in-person weekly with mission team members. At these weekly meetings, interns proposed and produced artworks about the Psyche mission, and they were exposed to subject matter experts working on the mission, to provide context and content. Produced artworks were shared through the mission social media accounts, exhibited to the public via an on-campus Showcase, and compiled into a coffee table book, which was made freely available via download to the public with printed copies sent to interns and mission leadership for outreach purposes.

Following its inaugural year, the Psyche Inspired program expanded nationally. The second cohort of Psyche Inspired (2018-2019), referred to as the Iron Class, was comprised of students from nine different universities in pursuit of 12 different majors, followed by the third year, referred to as the Cobalt Class, with 16 interns from 12 different universities in 18 different majors (including double majors). The Nickel Class, representing the fourth year of Psyche Inspired, included 15 interns from 8 different universities across the nation in pursuit of 15 different majors (including double majors). The Copper Class, representing the fifth year of Psyche Inspired, included 15 interns from 14 different universities in 13 different majors (including double majors). Through these first years of Psyche Inspired, participants from 38 colleges and universities (see Appendix C) have created more than 260 artworks to engage the public in the mission. These pieces and any subsequent works may be found in the Psyche Inspired gallery: https://psyche.asu.edu/galleries/artwork/.

#### Psyche Inspired Products

**Fig. 5:**
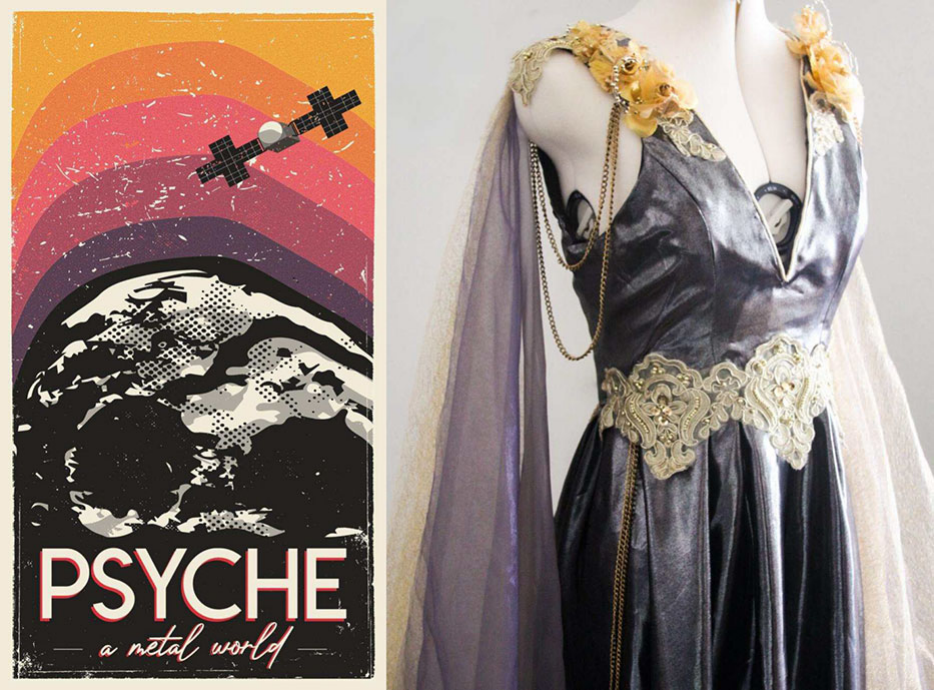
Psyche Inspired projects range from posters (L: Daniel Zepeda-Cuba) to a handmade dress (R: Siena Smania)

The primary products of Psyche Inspired are the artworks themselves (see e.g., Fig. 5, Fig. 8). Over the course of an academic year (September to May), each Psyche Inspired intern creates 4 creative works meant to communicate Psyche mission concepts to the public. In total, each cohort is responsible for the creation of more than 60 art pieces. Each piece is displayed, with accompanying interpretive text, on the interns’ individual webpages on the Psyche mission website, as well as highlighted on the @MissionToPsyche social media accounts throughout the year. They are further archived and disseminated through a coffee table book that displays each piece along with introductory front matter such as letters from the program’s student manager and the mission principal investigator and short descriptions of relevant mission content. The covers of these coffee table books are designed by either a member of the Psyche Inspired class or by a member of the Psyche Student Collaborations team. The books are made available to the public via a downloadable PDF on the Psyche mission website.

**Fig. 6:**
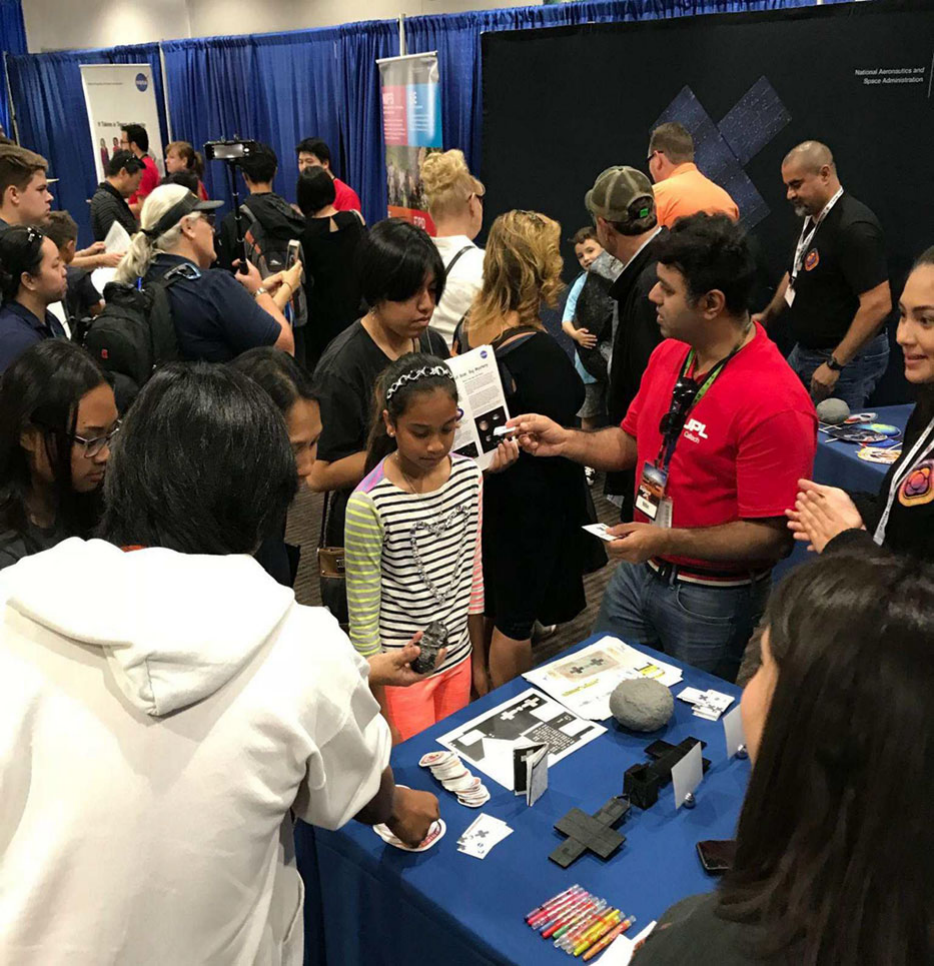
JPL Ticket to Explore (shown) and other public events locally and nationally are supported by Psyche mission team members, including science outreach interns

At the end of the academic year, Psyche interns have their works exhibited in an annual showcase. Originally, the showcase was held entirely in person but, as the Psyche Inspired program expanded to include national participants, it has evolved to incorporate a virtual component as well. This hybrid approach allows local communities to see a subset of works in person as well as appreciate the full catalog of art through online exhibits.

In response to the COVID-19 pandemic, the Psyche Inspired program transitioned its showcase to an entirely virtual format. Interns were interviewed in pre-recorded sessions to discuss their participation in the program. These interviews were made available on their respective web pages along with an opening introduction video by mission leadership. In addition to these recordings, the public was encouraged on social media or through a comment form to send in questions to the interns about their experiences within the program and their inspiration and interpretation of their art pieces. As appropriate, questions were distributed to the interns and intern responses were posted and used along with the pre-recorded interviews to create short videos to help promote the application for the next Psyche Inspired cohort.

The virtual showcase experience was enhanced by the use of a 3D gallery created on the free *ArtSteps* platform. With this platform, the public was able to walk around the virtual gallery and enjoy the pieces created by the interns by interacting with objects to read about both the pieces and the interns that created them. As possible, capstone or other undergraduates contribute to the showcase, such as an additional virtual experience created for the Cobalt Class Showcase through the involvement of a mobile app development class at ASU. Through this partnership, an app was designed to showcase the Psyche Inspired pieces, as well as highlight the nationwide distribution of interns within the Cobalt Class. For the Nickel Class, an ASU Public Relations capstone team aided in the development of promotional content and marketing plans for Psyche Inspired, as well as showcase design and implementation.

Psyche Inspired art pieces are also exhibited beyond their cohort year at public events (such as The Art of Planetary Science at the University of Arizona, ASU Earth & Space Exploration Day and Homecoming, and Phoenix Fan Fusion) and museums (such as the i.d.e.a. Museum in Mesa, AZ, the MOXI in Santa Barbara, CA, and the Phoenix Airport Museum in Phoenix, AZ). The works are cataloged and stored at ASU when not on exhibit at local, regional, and national events and spaces.

#### Feedback and Program Improvement

As the Psyche Inspired program has grown in its reach, structured vetting efforts of proposed artwork concepts have been implemented to ensure that Psyche mission content is accurately (while still creatively) represented in the artworks and accompanying interpretive text. This is implemented through three formal checkpoints during the development of each artwork: Once when the artwork concept is proposed, once at the halfway mark, and once at completion. At these checkpoints, the artworks are reviewed by program coordinators and the mission principal investigator. The Psyche mission content portrayed in the works and the written descriptions provided as interpretive text are reviewed for potential misconceptions, and feedback or editing is provided to the interns as appropriate. Art pieces are also reviewed and discussed informally during Psyche Inspired group meetings when interns are encouraged to share the status of their project with their cohort, further ensuring accurate portrayal and discussion of Psyche mission information throughout the creative process.

Beyond the feedback cycle between the Psyche Inspired interns and the program coordinators about the artwork, the coordinators implement program-wide improvements based on student feedback gathered through one-on-one check-ins throughout the year and a final group debrief following the Psyche Inspired Showcase. Additionally, Psyche Inspired has leveraged the talents of capstone teams participating in the Psyche capstone program. This interdisciplinary, inter-program approach allows the two programs and their participants to inform one another’s experiences. For example, in 2019, an applied engineering capstone team from Michigan State University (MSU) conducted a formal analysis of Psyche Inspired. They assessed the program timeline, joined Psyche Inspired meetings to engage with interns and observe the synchronous component of the internship, and conducted surveys and interviews with the interns. Their resulting recommendations (such as streamlined due dates, thematic project assignments, greater involvement by science, engineering, and arts experts, and requiring interns to complete the introductory *Process and Lifetime of a Space Mission* online course [see Sect. 3.1]) were implemented by the program with the subsequent cohort. In spring 2021, a public relations capstone team from ASU devised a marketing plan with creative methods for advertising and augmenting the year-end showcase, leading to the creation of a special Psyche mission Snapchat filter to boost social media presence, virtual gift bag items, and future plans for a Unity experience for users to view the art pieces in a more elaborate virtual setting. Other projects relevant to Psyche Inspired undertaken by Psyche capstone teams include an industrial engineering team that built an online artwork archiving system to keep track of the artworks, including temporary loans to exhibit spaces, and multiple computer science and software engineering capstone teams that have incorporated the art into their web-based game projects.

## Public Programs

Though all Psyche Student Collaborations programs are university- or undergraduate-based, many also include components that are available to the public, including lifelong learners worldwide and K-12 teachers and students, supported by undergraduate student participants.

### Innovation Toolkit – Online Courses

The NASA Psyche Mission Innovation Toolkit (https://psyche.asu.edu/get-involved/ innovation-toolkit/) includes a series of free online courses based on the real-world challenges and skills associated with the mission’s science, engineering, technology, and teamwork. The short courses are self-paced, may be started at any time, and may be taken as stand-alone courses or be completed as part of a sequence. Each course results in a certificate of completion and may be included on a participant’s resume or CV as “professional development.” Though targeted at early college level, through these courses lifelong learners worldwide can learn and practice a range of new information and skills critical for innovation - both technical and “soft” skills sought after by employers in diverse work environments.

Developed in collaboration with ASU’s EdPlus (digital education group) and student worker science writers, with the expertise of subject matter experts from the mission and other collaborators, the courses incorporate new and pre-existing content and materials, with the goal of presenting them in ways that are engaging and comprehensible to a wide audience. Topics alternate between science or engineering content (e.g., space mission life cycle and small solar system bodies) and general workplace skill or competency content (e.g., inclusive teamwork or troubleshooting strategies). Current and upcoming courses descriptions are outlined in Appendix H.

The courses are advertised via traditional and social media, flyers at public events, and via team member presentations. As of the end of the 2021-2022 academic year, more than 2,500 learners have enrolled in *The Process and Lifetime of a Space Mission* and almost 700 learners have enrolled in *Inclusive Mindset*. Upcoming courses include *Countless Worlds in Our Solar System: Asteroids, Comets, and Meteorites* and *Strategies for Troubleshooting Problems in Everyday Life and Work*. Additional courses will be developed throughout the lifetime of the mission.

### Science Outreach Interns and Docents

As with other university-based principal investigator-led missions, student workers, both undergraduate and master’s students (as well as summer students and other interns at partner institutions as described below) contribute significantly to Psyche Student Collaborations efforts. These students are employed as either science outreach interns (24 as of the 2021-2022 academic year) or docents (14 as of the 2021-2022 academic year), with associated responsibilities and opportunities. Doctoral-level science and engineering students and postdocs are employed by individual science co-investigators. Some participate with Psyche Student Collaborations as volunteers, but it is not a requirement of their involvement with the mission.

#### Intern and Docent Overview

Psyche science outreach interns directly support and co-manage the programs within Psyche Student Collaborations and associated public engagement efforts. They are hired for specific program needs and, with a wide range of majors and previous experience, they provide essential work and a fresh perspective. With mission team member guidance, the interns co-manage the Psyche capstone program, Psyche Inspired, the mission website, @MissionToPsyche social media, and school visits and public events. They develop and edit content for the Innovation Toolkit online courses, public outreach materials, and conference and journal publications. They provide support to Psyche capstone students and Psyche Inspired interns, helping with technical questions, programming, access to resources, review of materials, and administrative documentation.

Docents work for the ASU School of Earth and Space Exploration (SESE) and are partially supported by the mission to engage more than 10,000 K-12 students and teachers annually in giving tours of the Gallery of Exploration at ASU, including an introduction to the mission (this number was accurate as of the 2019-2020 academic year, prior to the pandemic, and is expected to return to pre-pandemic levels). The docents receive training from the Community Outreach, Education, and Public Engagement staff for SESE at ASU covering a variety of subjects, including robotic exploration, geologic and planetary sciences, and instrument assembly and testing. Their majors range from music and performing arts to communications to various engineering disciplines and the sciences, which enables them to connect in unique ways with diverse groups of K-12 students and teachers.

Psyche Student Collaborations has also benefited through the participation of NASA Space Grant Interns (as volunteers at events) and summer students (such as students participating in a Research Experiences for Undergraduates [REU] program or interns working with Co-Investigators at other institutions). Depending on their role, these students are onboarded through the standard Psyche Student Collaborations process and integrated into the team.

#### School Tours

Interested Phoenix-area schools can schedule an onsite guided tour of SESE’s Gallery of Scientific Exploration. During these tours, K-12 students and their teachers engage with a university student docent to learn about space exploration and the range of people contributing to it. Docents (predominantly undergraduates majoring in the sciences or engineering) are trained by educational, science, and engineering subject matter experts in ASU’s School of Earth and Space Exploration.

Participating school groups learn about the Psyche mission through discussions about differentiated bodies, meteorites, cratering, and the simulated experience of flying by the asteroid as a part of the 3-D astronomy show they attend. Each year since the mission’s selection, the reach of the school tours has grown by approximately 10%. The program reported its highest reach to-date in 2019 (pre-pandemic), engaging with over 10,000 students. The tour groups represent over 100 schools and 40 districts within Arizona. To date, the bulk of participating classes (95%) are between 5th and 8th grade. Those in grades 9-12 and homeschool groups make up an additional 4% and the remaining (1%) are from grades K-4th.

#### School Visits

For K-5th grade classes in the greater Phoenix area unable to visit the ASU campus, the Psyche outreach interns offer on-site classroom visits with the goal of exciting students about space exploration and the mission and allowing them to meet college students and ask questions about their education and career aspirations. The content of the classroom visits is tailored to the age group and focused on what the mission entails, why the team is interested in studying Psyche, and the Student Collaborations opportunities of the mission. During the 2019-2020 academic year, the intern team visited 10 schools and presented to approximately 400 students and teachers. As a result of the COVID-19 pandemic, school visits for the 2020-2021 and 2021-2022 school years were canceled, however, materials from the school visits are also shared on the mission website by grade band (https://psyche.asu.edu/get-involved/models-and-materials/) and were used for a number of virtual school-related events during the pandemic.

#### Public Events and Presentations

The science outreach interns, docents, and many members of the mission team from universities, NASA centers, and partner institutions participate in public events and presentations each year, and efforts are made to reach and inform a wide range of communities and stakeholders about the mission. This includes scientific and technical peers at major conferences (e.g., American Geophysical Union [AGU] Fall Meeting, American Institute of Aeronautics and Astronautics [AIAA], Institute of Electrical and Electronics Engineers [IEEE], Capstone Design Conference, Undergraduate Research Programs conference), large local public events (e.g., USA Science & Engineering Festival, Phoenix Fan Fusion, JPL Ticket to Explore, ASU Earth and Space Exploration Day, and the Cambridge Science Festival in Massachusetts), and smaller community organizations (e.g., astronomy clubs, school science nights). In the first four years since mission selection, the mission team has participated in hundreds of events reaching more than 100,000 attendees. Mission team members coordinate with the Student Collaborations team to report their participation details and to request materials or student staffing if needed.

## Outreach Products and Efforts

Psyche Student Collaborations interns and program participants formulate, create, implement, maintain, and disseminate many mission-related outreach products and efforts (e.g., the mission website, social media, videos, and public events and presentations) in close coordination with the Psyche Student Collaborations lead and Psyche mission team members. This helps extend the reach of the mission and the Student Collaborations program beyond the program participants themselves to many new audiences.

### Mission Website

The Psyche mission is represented by NASA (https://www.nasa.gov/psyche/), JPL (https://www.jpl.nasa.gov/missions/psyche/), and NASA Solar System Exploration (https://solarsystem.nasa.gov/missions/psyche/in-depth/). Although NASA’s webpage is considered the primary page, as with other principal investigator-led missions (e.g. Lucy: http://lucy.swri.edu/, OSIRIS-Rex: https://www.asteroidmission.org/, MAVEN: https://lasp.colorado.edu/home/maven/) the mission team also maintains https://psyche.asu.edu/ to provide extensive in-depth information about the science, engineering, timeline, and public opportunities related to Psyche and, once at the asteroid, will host processed and calibrated Imager data (see Sect. 5.3.4; raw uncalibrated images will be hosted at JPL). The website and other outreach products align to the Psyche Brand Guidelines, which serve “to establish boundaries and best practices in the use of the Psyche Mission badge, visual system, and appropriate copy [to]... ensure consistency and quality in all Psyche-related designs” (https://psyche.asu.edu/wp-content/uploads/2018/03/20200528_Psyche_BrandGuide-v2_6.1_20_rev-.pdf). Details about the Psyche mission website are provided in Appendix I.

### Social Media

Major mission milestones and highlights are shared via @NASASolarSystem on Twitter, Facebook, and Instagram (and occasionally amplified by other NASA accounts), which is managed by JPL. To share daily mission content, opportunities to get involved, and an inside look at the team, the mission uses @MissionToPsyche (formerly @NASAPsyche) on Twitter, Facebook, and Instagram, which is managed by a trained outreach intern with oversight from the Student Collaborations lead and the mission principal investigator. When appropriate, upcoming posts from @MissionToPsyche are coordinated with other mission partner accounts (such as @Maxar) to help amplify the content. As possible, the @MissionToPsyche account implements suggestions and content from communications-related capstone teams (such as recommendations from a Public Relations capstone team to make regular use of certain hashtags and platform-specific content, or recommendations for images and animations from a graphic design capstone team), mission personnel, alumni, and other stakeholders.

In particular, the @MissionToPsyche accounts allow the mission to acknowledge the many people and institutions involved. Special themed campaigns, such as #PsychePartner and #PetsofPsyche, highlight those elements. Additionally, on January 1, 2020, the Psyche principal investigator, with the encouragement of NASA Headquarters, started a new series of regular tweets with the tag #PI_Daily from her personal account (@ltelkins) and invited other NASA principal investigators and deputy principal investigators to do the same, in order to share the “chores, challenges, tasks, assignments, and duties” and “share the experience.” @MissionToPsyche regularly retweets her #PI_Daily posts (and occasionally reposts on other platforms) and maintains an archive on the mission website (https://psyche.asu.edu/mission/faq/pi_daily/). Details about the brand strategy and engagement for the @MissionToPsyche accounts are provided in Appendix J).

### Video Products

Videos constitute another series of projects created to build public awareness of, and engagement with, the Mission, as well as providing important introductory material for new Student Collaborations program participants being onboarded each fall. From the time of mission selection, the goal of the videos has been to tell the story of the mission and the people behind it in a series of short vignettes, which can be combined into a more extensive video documentary post-launch, after arrival at the asteroid, and when the mission is complete. An Arizona-based production company, True Story Films, was selected to be the official videographers for the mission, in addition to in-house videographers from NASA, JPL, and Maxar and cinematic illustrator Peter Rubin.

The Psyche mission team works together to determine the focus of each video, ensuring a diversity of team members, locations, subject areas, and perspectives. The ultimate goal of the collection of videos, beyond public awareness and engagement, is to highlight the diversity of background, knowledge, and skill needed for a successful space mission. Each video is relatively short, most are less than five minutes, making them easy to watch on YouTube, for public presentations, to share on social media platforms and press releases, for events, and other public outreach venues. Details about the Psyche mission video products are provided in Appendix K.

## Future Plans and Expanded Opportunities

As described above, the Psyche Student Collaborations program is inspired by and built upon the extensive foundation of public engagement, education, and outreach efforts and expertise of NASA and partner institutions. Below is presented a summary of existing (and potential) plans for Psyche Student Collaborations post-launch.

### Capstone

The Psyche capstone program will continue to grow in diversity of participating institutions, disciplines, and projects, if not in total number of teams participating annually (currently approximately 60 teams per academic year engaging more than 300 students). Goals informed by feedback from participants and participating institutions include engaging more interdisciplinary teams, developing targeted professional development to help students improve teamwork and communication, creating more casual networking opportunities between students and alumni and Psyche team members, providing archived lectures as podcasts to facilitate access for students who cannot attend synchronously, and increasing interim opportunities for students to get direct feedback on their projects from subject matter experts. Additionally, engaging capstone teams (such as those in applied engineering, engineering management, graphic design, and public relations) in helping to improve aspects of other Psyche programs (described below) has been fruitful and is an area planned for expansion, as possible.

### Psyche Inspired

In the future, Psyche Inspired will continue supporting undergraduates in creating artworks about Psyche in collaborative ways that invite the public to participate with the pieces made by the interns. Through displays on their campuses, exhibits at regional museums, and opportunities to engage with members of existing NASA programs such as the Museum & Informal Education Alliance and Solar System Ambassadors, the Psyche Inspired interns and their artworks will be able to reach a wide audience interested in STEAM. Artworks may continue to be displayed at venues such as the MOXI STEAM Museum in Santa Barbara, CA, the Sky Harbor Airport Museum in Phoenix, AZ, the Visitor Center at NASA’s Jet Propulsion Laboratory, and at other NASA venues. Expansion of social media presence is planned through the integration of strategic promotional timelines and more interactive means of spreading the word (e.g., filters or lenses on Instagram and Snapchat, Instagram and Facebook Live events, swipe up options for informational links, etc.). Further development and implementation of the showcase gallery enhancements as recommended by capstone team collaborators will strengthen the virtual component of the showcase. These include offering immersive, virtual campus backgrounds from each of the institutions the interns attend, expansion of the virtual galleries to include interactive 3D YouTube videos and gamified experiences, and a forum for guests to ask interns questions about their art pieces and experiences in the program.

### Public Engagement and Partnership Opportunities

The energy, enthusiasm, creativity, and relatability of undergraduate students engaged in Psyche Student Collaborations as interns, participants, and program alumni offers a renewable resource that facilitates the program’s efforts to bring as many people as possible along on the journey to a metal world. With their help, and building on the history and experience of NASA’s extensive portfolio of programs, subject matter experts, and public engagement, public affairs, and media relations professionals, the mission is able to efficiently implement a number of public opportunities based on successful prior models. The implementation of many of these opportunities also benefits from analyses and reports produced by Psyche capstone teams, as described below.

#### Pre-Launch and Launch Coverage

NASA and JPL provide support to missions for public engagement and media coverage leading up to and including launch, through efforts such as the development of fact sheets and press kits, coordination of press briefings and NASA Socials (https://www.nasa.gov/connect/social/index.html), and live launch coverage on NASA TV and NASA social media channels. Recently, commercial spaceflight companies have conducted similar efforts with their own launches and may offer other ideas for the mission to consider. To help prepare for the most comprehensive and compelling launch lead-up and coverage possible, during the 2021 spring semester a team of capstone students in applied engineering at Michigan State University (MSU) conducted research and a comparative analyses of lead-up events, coverage, promotion, and public participation opportunities of recent launches by NASA, SpaceX, and other commercial space entities and created a customizable cause and effect matrix to help the mission team explore what would be likely to be the most effective in terms of engaging wide audiences. They also provided recommendations for opportunities the mission could incorporate to help gain substantial public attention and generate excitement and participation. Results from this capstone project, particularly the cause-and-effect matrix, may be used to inform the design and implementation of mission launch lead-up.

#### Beam Your Name to Psyche

Many NASA missions have conducted successful “Send Your Name” public campaigns in which members of the public worldwide submit their names via websites, receive a certificate, “boarding pass,” or other acknowledgement of their participation, and have their name included on a chip or plaque on a spacecraft (e.g., https://mars.nasa.gov/participate/send-your-name/, http://parkersolarprobe.jhuapl.edu/The-Mission/Name-to-Sun/, http://lro.jhuapl.edu/NameToMoon/, http://lasp.colorado.edu/maven/goingtomars/send-your-name/). The mission is investigating a similar opportunity, *Beam Your Name to Psyche*, with the goal highlighting the way the mission team communicates with the flight system via the Deep Space Network and, potentially, through the Deep Space Optical Communication (DSOC) technology demonstration (see e.g., NASA’s Laser Communications Relay Demonstration public campaign: https://www.nasa.gov/mission_pages/tdm/lcrd/participate-in-lcrd-first-light.html). As an early step in determining the preparation and resources needed to support the collection of names, during the 2019 spring semester an applied engineering capstone team from MSU researched the processes, work flows, data management needs, and public interest of previous opportunities, including interviewing subject matter experts from Mars Public Engagement (*Send Your Name to Mars*) and surveying members of the public about preferences related to a potential “Send Your Name” registration experience. In the 2020 fall semester, a public relations capstone team from ASU conducted an audit of previous “Send Your Name” campaigns and provided suggestions for language, graphics, and promotion of a similar Psyche opportunity. The results from these two capstone projects will help guide the development of “Beam Your Name to Psyche.”

#### Asteroid Appearance Competition

**Fig. 7:**
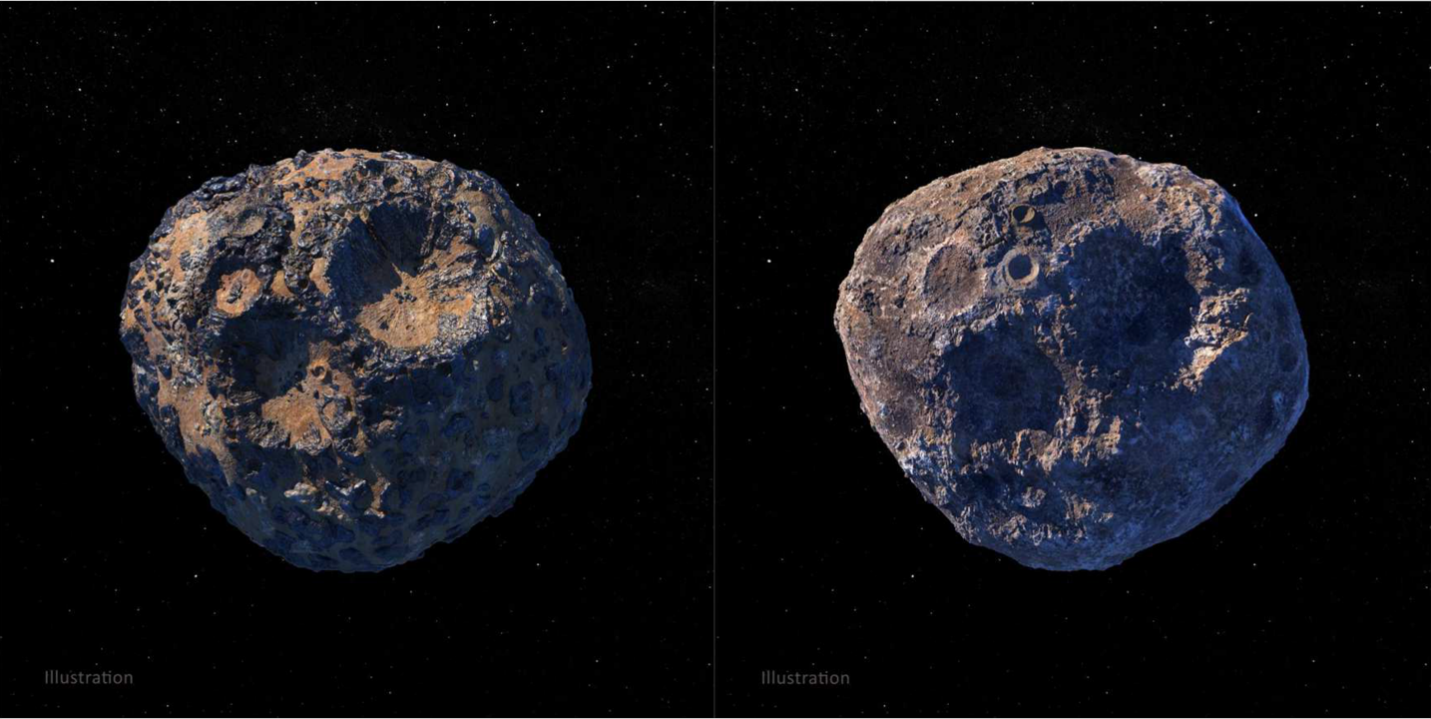
Scientifically-informed artist’s illustrations. Based on data obtained from Earth, scientists believe Psyche is a mixture of metal and rock. The rock and metal may be in large provinces (L) or intimately mixed on a scale too small to detect from orbit (R). Image credit: NASA/JPL-Caltech/ASU

**Fig. 8:**
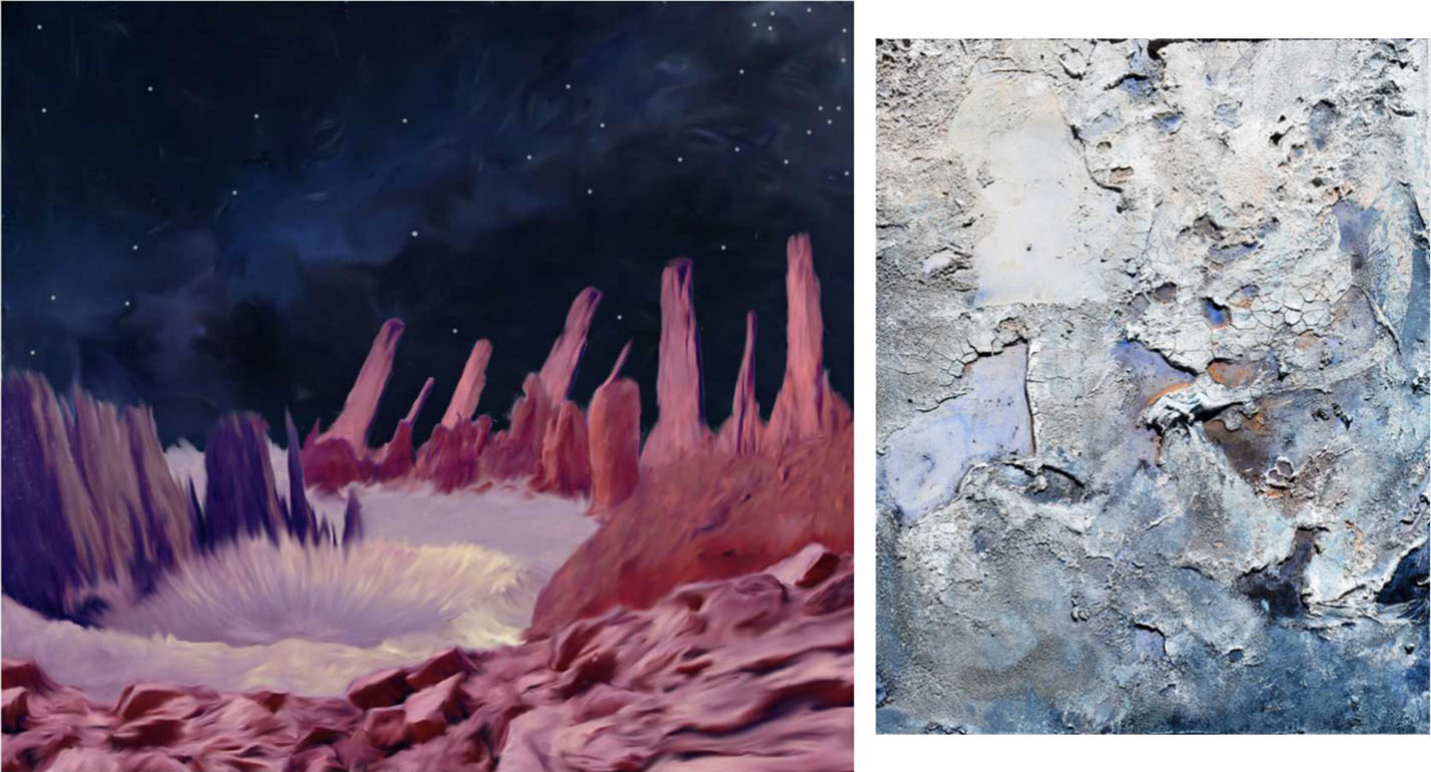
Creative interpretations by Psyche Inspired interns Finn Witt (L) and Zarina Karapetyan (R)

One of the challenges of engaging the public in the Psyche mission is that there are no images of the Psyche asteroid, and the earliest images of it will be obtained as the flight system arrives at its target. A series of scientifically informed artist’s renderings (see Fig. 7) and the imaginings of the Psyche Inspired interns (see e.g., Fig. 8) provide tantalizing possibilities.

The truth, however, is that no one can know what Psyche will look like, and that presents an opportunity to invite the public to imagine Psyche along with the team. Informed by successful NASA projects such as *Cassini Inspires* (https://saturn.jpl.nasa.gov/mission/cassiniinspires/) and *Imagine Ceres* (http://dawn.jpl.nasa.gov/DawnCommunity/imagine_Ceres_gallery.asp), during cruise the public would be invited to submit their creative ideas in different media categories (such as drawing, painting, ceramic, Lego, Minecraft, animation) as well as different categories for students, for professional artists, for enthusiasts, etc. After inspection, the creative works would be put online for general review, and then winners announced after the flight system has arrived at Psyche and images are released. To be informed of best practices for such opportunities, during the 2021 spring semester a team of capstone students in applied engineering at MSU conducted research and analysis of the various creative contests and competitions NASA has hosted in the past to compare the procedures and rules, how they have been promoted, what the outcomes have been, and what has been effective in engaging the public in order to make recommendations about what the mission could do to make the effort stand out and reach a wide audience. The findings from this capstone project will be used to finalize the design of the asteroid appearance competition.

#### Multispectral Imagers Data Direct to Public Websites

Following the tradition established by many previous NASA Solar System exploration missions, including principal investigator-led missions (e.g., Dawn: https://solarsystem.nasa.gov/missions/dawn/galleries/images/, InSight: https://mars.nasa.gov/insight/multimedia/raw-images/, Lucy: https://svs.gsfc.nasa.gov/Gallery/Lucy.html, and OSIRIS-Rex: https://www.asteroidmission.org/galleries/spacecraft-imagery/), the Psyche team plans to share raw and derived data products with the world as quickly and openly as possible. The aim is to increase awareness and excitement about the mission by inviting the public to be among the first to see each new spectacular scene coming from the mission’s exploration of this enigmatic asteroid. Specifically, raw images from the Psyche Multispectral Imagers (“the Imagers”) will be automatically created after each downlink and then rapidly and automatically posted as PNG-format image files on a publicly accessible web site hosted by JPL. Based on experience with similar JPL image distribution systems used for images from the Spirit, Opportunity, Curiosity, and Perseverance rovers (e.g., https://mars.nasa.gov/mars2020/multimedia/raw-images/), for example, the Psyche team expects to be able to post raw images for public viewing within only seconds to perhaps minutes after each set is received and processed by JPL and NASA’s Deep Space Network.

With oversight from the Imager lead and his team, science outreach interns will help manage a second publicly accessible web site, hosted at ASU and part of ASU’s overall mission web page structure, that will host additional processed and calibrated Imager data. These additional images will include calibrated individual monochrome images, natural color and false color images made by blending data from various combinations of the Imager’s seven different color filters, monochrome and color mosaics of specific regions of geologic interest, and eventually, global monochrome and color mosaics of the entire surface. The team envisions that these products will be made rapidly available when it is possible to do so (for example, within a few hours of downlink for calibrated individual image products), but other products (such as large regional mosaics or global maps) might take days, weeks, or months to complete for posting. As with other NASA missions, all of the Imager calibrated and derived image products will also be available through the Planetary Data System (PDS), which is the official repository for all science data products. Nonetheless, the intention is that the ASU Imager website will be a user-friendly long-term source for highest-resolution versions of the many kinds of derived image products that the team plans to share with the world from this exciting mission of exploration. This resource may also be a source of inspiration for new capstone projects and for cohorts of Psyche Inspired interns in the future.

#### Engagement with NASA’s Solar System Ambassadors and Museum & Informal Education Alliance

NASA has existing and robust programs involving enthusiasts, public engagement professionals, and informal learning venues in communicating with the public about space exploration, for example the Solar System Ambassadors program (https://solarsystem.nasa.gov/solar-system-ambassadors/directory/) and the Museum & Informal Education Alliance (https://informal.jpl.nasa.gov/museum/). In Phase B and early in Phase D of the mission, team members gave professional development presentations about the mission to NASA’s Solar System Ambassadors and members of the Museum & Informal Education Alliance (formerly the Museum Alliance) and additional presentations will take place pre- and post- launch and arrival. Additionally, relevant materials developed by the team, including the Psyche science outreach interns, Psyche Inspired interns, and capstone teams, are available to be shared with these groups. In particular, capstone-developed printable models, WebXR experiences for mobile phones, Virtual Reality (VR) experiences, and web-based games are available to disseminate to interested members of both groups. Other materials (classroom activities, videos, demonstrations, etc.) are also available for members of those groups who wish to present about Psyche to their local communities.

### Other Possible Public Opportunities

A benefit of Psyche’s status as a principal investigator-led mission based at a large university is that the talents, ideas, and enthusiasm of large numbers of undergraduate and graduate students, staff, and faculty inspire and enable the implementation of a wide range of public opportunities. Ideas are also shared by Psyche team members at other institutions. Possible student and public opportunities include:

- **“Hi”-ku from Psyche**
  In 2013, NASA’s Mars Atmosphere and Volatile EvolutioN (MAVEN) orbiter mission invited the public to submit a haiku about Mars for possible inclusion on the spacecraft https://lasp.colorado.edu/maven/goingtomars/send-your-name/haiku-with-maven/ In a nod to that previous competition, the “Hi”-ku from Psyche opportunity would give members of the public the chance to submit a haiku to say “hi” to the MAVEN orbiter during the Psyche spacecraft’s Mars fly-by during interplanetary cruise.
- **Deep Space Optical Communications (DSOC) Observing Opportunity**
  The mission will be carrying the Deep Space Optical Communication (DSOC) technology demonstration, which will demonstrate high-rate data transmission to Earth by shining a laser to a telescope at the Palomar Observatory in California. This signal also will be observable in the southwestern U.S. and, at times, beyond. With partners at NASA’s Deep Space Network, the mission is assessing the feasibility of engaging observers (whether other observatories or serious amateurs) in helping to track the signal to provide data to aid in pinpointing the position of the flight system and assessing the performance of the laser. A related effort could involve public viewing parties and “Psyche Nights” coordinated with amateur astronomy clubs, universities, and NASA’s Night Sky Network (https://nightsky.jpl.nasa.gov/).
- **Citizen Science: Crater/Surface Characterization**
  NASA has significant citizen science resources and infrastructure available to the public to engage with space missions (https://science.nasa.gov/citizenscience). As the Psyche mission is the first flight system to visit a body made largely of metal, there is significant uncertainty about the composition and appearance of the asteroid, including what geologic features may be found on the surface. Although the Psyche team has undertaken analyses and experiments to explore the possibilities of cratering on a metal-rich body (described in https://psyche.asu.edu/2019/09/05/simulating-asteroid-collisions-and-making-craters/ and in Marchi et al. 2020), upon arrival there will be extensive characterization activities needed. One possibility is to engage “citizen scientists” in characterizing craters (and possibly other surface features) in images of the surface, as has been done for other planetary bodies (e.g., NASA Citizen Science projects listed at https://science.nasa.gov/citizenscience, such as Planet Hunters TESS and Disk Detective, projects listed on the Planetary Science Institute’s CosmoQuest site https://cosmoquest.org/x/ and on Zooniverse https://www.zooniverse.org/; see also Fortson 2021). During the 2020-2021 academic year, an interdisciplinary team of ASU capstone students from computer science and engineering management, along with an astronomy and planetary science undergraduate from Northern Arizona University, investigated new automated crater identification techniques as a first step to planning for potential hybrid (computer/human) crater and surface characterization citizen science activities related to Psyche.
- **Crochet Psyche**
  A community with the potential to be engaged directly with planetary science is the extensive network of crafters around the country. The pre-flight conception of Psyche, with the potential for spires or flaps of metal, intricately mixed materials of different color and texture, and possibility of large fractures due to cooling of a metallic core yields visions of a stark landscape unlike anything on Earth. Yet the potentially angular forms are not dissimilar to those seen on the seafloor, particularly where reefs rise above the shallow ocean floor, teaming with spiky coral of every imaginable shape and color. The Institute of Figuring developed the *Crochet Coral Reef Project* (https://ocean.si.edu/oceanlife/invertebrates/when-art-meets-science-hyberbolic-crochet-coral-reef) to allow crafters to contribute to a reef-scale complex that has been exhibited at, among other places, the Smithsonian’s National Museum of Natural History. The myriad of shapes, textures and colors on Psyche could provide a comparable opportunity to allow crafters to engage in the science of Psyche by crocheting individual features (e.g., craters, metal spires, flaps, offset cracks) that, when combined, could be exhibited together in an interesting and creative expression of Psyche’s unique surface.

## Conclusions

In FY20, NASA had 1,861 total undergraduate interns (NASA OSTEM 2020), an opportunity open only to U.S. Citizens (https://intern.nasa.gov/) or, in the case of NASA’s $I^{2}$ internship program, open to citizens of the 15 countries that have an agreement with NASA (https://www.nasa.gov/stem/international-internships-for-students.html). The 5% acceptance rate for NASA internships (Anderson 2022) and roughly 400,000 STEM bachelor’s degrees conferred annually in the United States (Hussar et al. 2020) (even if only a subset of them are interested in working in space exploration) indicates that the demand for available NASA internships exceeds supply. With more than 300 undergraduates participating actively with the Psyche mission for a full academic year through capstone projects and arts and science outreach internships, and with no citizenship requirement (as long as the student is enrolled full-time in a U.S. institution), Psyche Student Collaborations demonstrates that individual mission programs, whether PI-led or flagship, have the potential to significantly increase this capacity to help meet demand.

By leveraging extensive engagement by undergraduates at all levels of Psyche Student Collaborations, and working within existing academic structures such as senior capstone courses, the component programs benefit from a cost-effective, renewable, and regenerative source of creativity, talent, and enthusiasm. Involving undergraduates in all phases of the program also supports the development of the next generation of explorers, contributes to the nation’s workforce preparation, and complements NASA’s existing undergraduate offerings by providing long-term opportunities for students to participate with the mission through established postsecondary education structures like capstone courses. PI-lead missions not headquartered at a university may wish to consider engaging university partners in their Student Collaborations planning in order to facilitate these opportunities (such as the case with the Lucy mission’s partnership between the Southwest Research Institute and ASU). Additionally, when selecting opportunities to implement, missions may wish to consider the intensity of resources, such as required mission team member involvement and the number of people who can participate in each opportunity (such as in Fig. 1 above), in order to develop a diverse program that balances breadth and depth. This includes designing opportunities so that interested mission team members, whatever their subject matter expertise, may contribute at a level that is comfortable and realistic for them. Finally, in selecting opportunities for inclusion in a comprehensive Student Collaborations program, remaining grounded in the core values and vision of the mission team and the participating institutions can help ensure that the program is considered, and supported as, an integral component of the mission as a whole.

## Figures and Tables

**Fig. 9:**
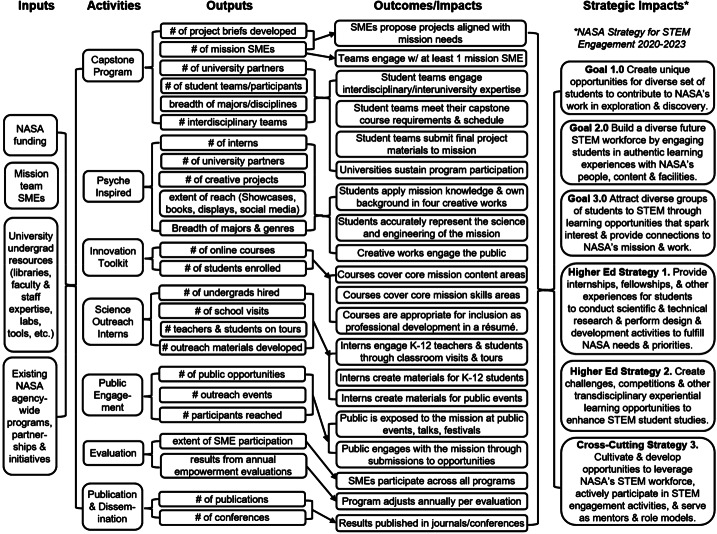
Psyche Student Collaborations logic model

**Fig. 10:**
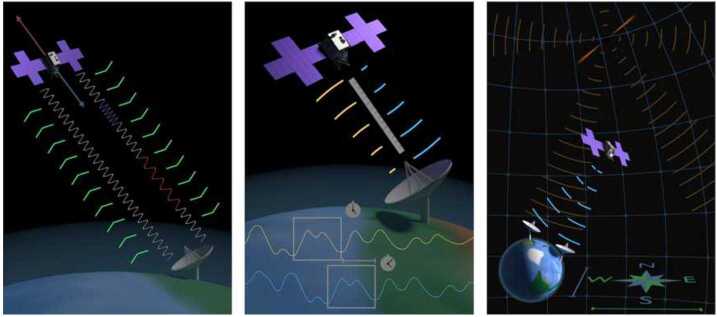
“Navigation Data Types Triptych,” a digital media piece created by Psyche Inspired alumnus Ben Conway for the Psyche project team during a JPL internship. Image Credit: NASA/JPL-Caltech

**Fig. 11:**
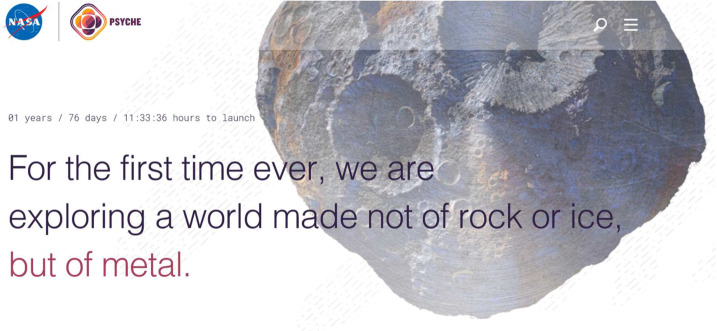
The Psyche mission website (https://psyche.asu.edu)

**Fig. 12:**
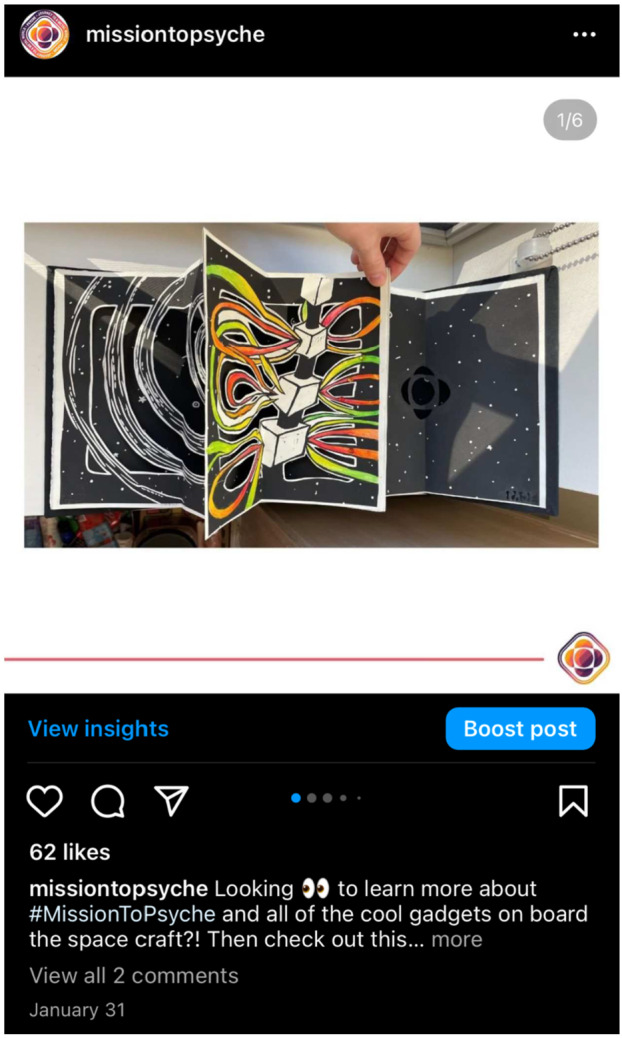
Example of the new @MissionToPsyche Instagram theme

## Data Availability

All Psyche Student Collaborations public material is available at https://psyche.asu.edu.

## Appendix A: Logic Model

The logic model (Fig. 9) depicts the relationships between the program inputs (the “funding sources and resource streams that provide support to the project”), activities (the “services, materials, and actions that characterize the project’s thrusts”), outputs (“products of these activities or a count that describes the activity”), and outcomes and impacts (the “the changes that occur as a result of the activities; they may be short or longer term”) (Fretchling 2010, p. 17; see also Friedman 2008). The final column, strategic impact, lists the specific goals and strategies from the NASA Strategy for STEM Engagement 2020-2023 are addressed by the Psyche programs (NASA 2020).

## Appendix B: Additional Proposed Student Collaborations Projects (Not Selected for Implementation)

As described in Sect. 1.1, NASA’s long history of educational and public engagement programs provided significant inspiration for the development of the Psyche Student Collaborations program. The list below provides an archive of additional proposed elements of Psyche Student Collaborations that were not selected by NASA for implementation.

### Planetary Science Summer Seminar (PSSS) Feeder Program

To develop a program to complement PSSS (https://pscischool.jpl.nasa.gov/), acting as an entry point for students from community colleges and non-Research One institutions.

### Tracking and Gravity Science

To partner with the NASA/JPL Goldstone Apple Valley Radio Telescope Project (GAVRT) to engage high school, community college, and undergraduate students in two Psyche-related observing campaigns to compare with Psyche team data (tracking the spacecraft trajectory and modeling the asteroid’s gravity field), modeled on similar previous campaigns (http://www.lewiscenter.org/Global-Programs/GAVRT/About/).

### CubeSat Summer School

To create and implement a new annual summer school at LANL, JPL, and ASU on CubeSat mission design or space hardware design and development to be planned and executed with members of the ASU School of Earth and Space Exploration, the Psyche team, JPL, and LANL.

### Remote Team Building and Competition

To carry out student competitions open to teams assembled anywhere in the world, modeled on NASA’s Space Apps Challenge (https://2017.spaceappschallenge.org/), with opportunities to conduct substantive work related to their expertise that can be listed on a resume or CV.

### Participating Early Career Scientist Program^1^.

As a complement to NASA’s existing participating scientist opportunities, an open call to scientists within five years of their PhD to join the science team for periods of one or two years each, to learn the running of the team, the process of a mission, to produce some research, and to meet and become integrated into the mission community.

## Appendix C: Psyche Student Collaborations Participating Institutions

**Table 4:** Psyche Student Collaborations Participating Institutions (through 2021-2022 academic year)

	Capstone	Psyche inspired
Albion College		•
Arizona State University - Downtown Campus	•	
Arizona State University - Main Campus (Tempe)	•	•
Arizona State University - Online	•	•
Arizona State University - Polytechnic Campus	•	
Boston University		•
Bowdoin College		•
Brown University		•
California College of the Arts		•
California State University - Los Angeles	•	
Caltech		•
Chaffey College		•
Cleveland State University	•	
Columbia University		•
Columbus College of Art and Design		•
Creighton University		•
Emory University		•
Florida A&M U-Florida State University College of Engineering	•	
Florida State University - Panama City	•	
Georgia Tech		•
Illinois State University		•
Indiana University-Purdue University Indianapolis (IUPUI)		•
Kansas City Art Institute		•
Lawrence University		•
Lehigh University		•
Michigan State University	•	
Michigan Technological University	•	
Morgan State University		•
Northern Arizona University	•	
Pasadena City College		•
Penn State	•	
Penn State - Behrend	•	
Phoenix College		•
Rochester Institute of Technology	•	•
Seattle University	•	•
Temple University		•
The Citadel		•
Trinity University		•
University of Arkansas	•	
University of California, Berkeley		•
University of California, Santa Barbara		•
University of Colorado – Colorado Springs		•
University of Florida		•
University of Illinois – Chicago	•	
University of Maryland		•
University of Michigan		•
University of Minnesota		•
University of Pittsburgh		•
University of Southern California		•
University of Texas – Tyler	•	
Utah State University		•
Virginia Commonwealth University	•	•
Virginia Tech		•

## Appendix D: Participant Undergraduate Majors

**Table 5:** Undergraduate majors (n = 1221)

	Students
*Arts*	(n = 61)
Graphic Design	35
Other (Animation, Illustration, Music, etc)	26
*Communications & Journalism*	(n = 23)
*Engineering*	(n = 947)
Aerospace Engineering	35
Applied Engineering	29
Biomedical, Chemical, Materials Science	21
Computers/Programming	
Computer Science	343
Computer Systems Engineering	59
Informatics	20
Information Technology	14
Software Engineering	92
Electrical Engineering	82
Engineering Management	61
Industrial Engineering	10
Mechanical (including Robotics)	180
*Natural Sciences (Astrobiology, Astrophysics, Biology, Geoscience, Physics)*	(n = 61)
*Other (e.g. business, forensics, interdisciplinary studies, public policy, etc.)*	(n = 129)

## Appendix E: Alumni Class Names

**Table 6:** Alumni class names to date

Academic year	Class name
2017-2018	Titanium
2018-2019	Iron
2019-2020	Cobalt
2020-2021	Nickel
2021-2022	Copper

## Appendix F: Student Artist Contributions to Project Development

The work of Psyche Inspired interns engages the mission team as well as the public. While the most memorable end results of NASA’s space program certainly include footprints on the Moon and breathtaking images of celestial objects, NASA’s successes begin with blank pages and screens upon which tough technical analyses must be conducted by both scientists and engineers. Though there may seem to be little room for art in serious space exploration, none of NASA’s successes could have come about without the imagination of its team members. The imagination is the genesis of all flight projects, and imagination is made into reality through visualization, which can and often must include art. Likewise, some of NASA’s most difficult moments can and have been traced to what might be categorized as a failure of imagination, where a key piece of data, presented and visualized in the right form, could have made the difference. No space fan who was alive in 1986 will forget Nobel Laureate and Caltech Professor Richard Feynman dropping a sample of the Space Shuttle O-ring into ice water to demonstrate its loss of resilience at cold temperatures. Though not necessarily a work of art, the principle is the same: showing, not telling, has power.

There is a place in space exploration for artistry, which can go beyond inspiration and make real contributions to the advancement of space missions. Mission project members spend a great deal of time plotting data and showing the geometry of their spacecraft and its environment in ways that can easily be understood. It is critically important to create visualizations of how operations may work and accurately show these results to other project members who are trying to solve tough technical problems and need to understand what they are viewing. Effective visualization techniques can contribute to efficiently communicating complex topics.

As an example of the importance of visualizations, Psyche (the asteroid) rotates at an angle of nearly 90° to the plane of the planets, spinning “on its side.” For the mission to succeed, a fast-orbiting spacecraft must fly in a variety of orbits and make measurements of a fast-spinning asteroid, all while avoiding too-long eclipses (when the spacecraft is behind the irregular-shaped spinning body) while both the spacecraft and the asteroid orbit around the Sun resulting in changes in surface lighting and eclipse zones over time. Even the most experienced rocket scientists and engineers may struggle to visualize this scenario in their minds, but it can be depicted- including portraying critical estimations of the required science coverage to meet mission goals - through visualization and art.

The Psyche project has already directly employed, as JPL interns, members of the Psyche Inspired program to produce graphic designs to effectively describe its mission. These designs have helped provide quick-glance depictions of the mission goals, telecommunications, launch and orbit geometry, navigation techniques, physics of electric propulsion, and optical communication for team members that might not be specialists in a specific area (Fig. 10). Furthermore, the Psyche Mission Plan - arguably the most widely read project-level document - is graced with over a dozen works from the Psyche Inspired collection to date (with more coming in future releases).

## Appendix G: Psyche Inspired Application FAQs Provided to Applicants

From https://psyche.asu.edu/get-involved/psyche-inspired/psyche-inspired-application- 2022/

### What Will Psyche Inspired Interns do?

Psyche Inspired interns will use their creative talents to share, explain, and highlight the excitement, innovation, and scientific and engineering content of NASA’s Psyche mission. They will become part of the Psyche team, with the opportunity to interact with Psyche scientists and engineers from NASA, ASU, and other partner institutions, and with other Psyche Inspired interns and staff.

### What do We Expect from Our Psyche Inspired Interns?

Psyche Inspired interns will *meet once a week via videoconferencing* with staff from Psyche Inspired and the mission team. The meetings are required and will be held on Fridays from 12 p.m. to 1 p.m. Pacific Time.^2^

Psyche Inspired interns will *create at least one* original *creative work every six weeks* during the 2022-2023 school year, which will amount to four works throughout the school year. These works will be guided by the mission themes of science, technology, engineering, mathematics, exploration, inspiration, teams, and inclusion. Proposals, drafts, and final products will be provided to the Psyche Inspired staff for review and then will be posted to, and highlighted on, the Psyche mission website hosted by ASU (https://psyche.asu.edu), blog, and/or social media accounts. In late spring 2023, these works will be curated into an online showcase with distributed campus participation (if possible) and then incorporated into a coffee table book.

Psyche Inspired interns will take the Psyche online course, *The Process and Lifetime of a Space Mission*.

Psyche Inspired interns will follow NASA’s Image Use policies and other guidelines (which will be distributed by the Psyche Inspired staff upon selection).

Psyche Inspired interns will sign an intellectual property agreement with ASU prior to joining the program.

Psyche Inspired interns will be enthusiastic ambassadors for the Psyche mission, striving to inspire and excite the public through their creative work!

### How do Psyche Inspired Interns Benefit?

Psyche Inspired interns will be supplied with a stipend of $300 per intern to purchase materials needed to create their works (art supplies, computer software, crafting materials, etc.). The program will pay for completed physical works to be shipped to the project office at Arizona State University.

Psyche Inspired interns will have their work highlighted on the official Psyche mission website, blog, and/or social media accounts at least once per project.

Psyche Inspired interns will have their work exhibited in an online showcase with distributed campus participation (if possible) in late spring 2023.

Psyche Inspired interns will have their work showcased in a printed coffee table book and receive a copy of the book.

Psyche Inspired interns will have their work archived in an online portfolio.

Psyche Inspired interns may have their work presented and highlighted in Psyche presentations and exhibited at conferences, libraries, Psyche partner universities, NASA Centers, and at public events.

Psyche Inspired interns will gain knowledge and skills that may benefit them in their education and future employment. They will learn about the science and engineering of a space mission as well as gain skills and experience developing and promoting their own creative work locally and virtually.

Psyche Inspired interns will meet other creative students from around the country and will have the opportunity to share ideas and feedback and to collaborate.

### What Are the Eligibility Requirements of Psyche Inspired?

To be eligible, an applicant must:

- Be a current full-time enrolled undergraduate student at a university or community college in the United States and its territories and must have completed at least two semesters/terms at your current university or community college prior to applying. This will be confirmed with your institution before you are admitted to the program.
- Not be graduating any earlier than the end of the spring semester 2023 (nominally no earlier than May 2023).
- Commit to creating four original creative works over the period of the program (approximately one work per six weeks through May 2023).
- Be willing to sign an intellectual property agreement with Arizona State University.
- Commit to sending any physical works to the Psyche Inspired project office at Arizona State University (prepaid shipping labels will be provided).
- Commit to attending the weekly required meeting via videoconferencing on Fridays at 12 p.m. (noon) Pacific Time through the entirety of the program (through May 2023).
- Commit to responding to all communications from the program via email, text, or Slack (or similar) in a timely manner (within 48 hours).

Applicants are **not** required to be U.S. citizens.

### What Creative Genres May Apply?

We encourage any eligible undergraduate to apply who thinks that his/her creative medium would be effective at sharing the Psyche mission with the public. Examples include (but are not limited to):

3D physical works (such as ceramics, welding, glass blowing, fiber, jewelry, sculpture, etc.)

3D virtual works

Ads/posters

Comics/Animation

Choreography

Dance

Design

Drawing/sketching

Fashion/textiles

Film (including documentary)

Folk arts/crafts

Game design

Graphic art/design

Infographics

Media art

Music composition/performance

Painting

Performance arts

Photography

Poetry

Short story (fiction, nonfiction, science fiction, etc.)

Sonification (take information and turn it into sound)

Spoken word

Tattoo artistry

Theater

Other ways of creatively expressing mission information

### Is This Program Only for Art Majors?

No. Psyche Inspired is open to all talented, creative full-time enrolled undergraduate students at universities and community colleges in the United States or its territories, regardless of major.

Previous Psyche Inspired interns have come from a variety of majors, including aerospace engineering, animation, art & design, art history, astrobiology, astronomy, biochemistry, biogeoscience, biology, biomedical engineering, ceramics, chemical engineering, civil engineering, communication, computer graphics technology, computer science, creative technologies, digital culture, drawing, electrical engineering, engineering management, English, environmental sustainability management, forensics, geology, graphic design, graphic information technology, illustration, industrial design, interdisciplinary studies, jewelry/metalworking, kinetic imaging, mass media, materials science, mathematics, mechanical engineering, molecular environmental biology, music, new media design, painting, physics, public service and public policy, and sculpture.

### How Many Participants Will Be Selected?

We will select up to 16 interns for the 2022-2023 academic year: Up to 15 will be selected from institutions nationwide. At least 1 will be selected from Arizona State University.

### Who Reviews the Applications?

A committee comprised of Psyche mission team members and Psyche Inspired staff will review the applications and make the final selection.

### What Is the Process for Selection?

The goal of Psyche Inspired is to communicate the excitement, innovation, and scientific and engineering content of the Psyche mission through creative/artistic expression. To this end, we aim to include the best and widest range of creative genres and presentation styles. Because we invite an open range of creative pursuits, the criteria for selection are based on qualities that all selected interns should share:

- *Quality of example works* (Is the work executed with high quality and care? How creative/original is the work? Is the reviewer excited at the thought of seeing more work like this? Does the reviewer think this type of work would be appropriate for communicating the Psyche mission?)
- *Stated reason*($s$) *for wanting to be part of Psyche Inspired*.
- *Stated reason*($s$) *applicant believes he*/*she is a good candidate for Psyche Inspired*.
- *Demonstrated ability to follow through with projects*.
- *Demonstrated ability to engage with their local community or general public*.
- *Contact of references* (If deemed necessary, the selection committee will contact the applicant’s reference to discuss the qualifications of the applicant.)
- *Confirmation of eligibility* (If deemed necessary, the selection committee will contact the registrar or major department of the applicant’s university or community college to confirm enrollment.)

## Appendix H: Innovation Toolkit: Free Online Course Descriptions

From: https://psyche.asu.edu/get-involved/innovation-toolkit/

- *Course 1: The Process and Lifetime of a Space Mission*: A space mission encompasses so much more than reaching a destination and sending back discoveries! The Process and Lifetime of a Space Mission course shares the story of a team creating a NASA robotic space mission. As a participant in this course, you will learn about and practice elements of the process and lifetime of a space mission, from idea to flight. Aspects of the preparation and submission of a proposal, team building, design, construction, modeling, testing, launching, tracking, and data collection and analysis processes will be covered.
- *Course 2: Inclusive Mindset: Tools for Building Positive Team Culture*: Organizations, universities, and businesses (and missions!) benefit most when people work together smoothly as a team. An inclusive mindset, collaborative team culture, teamwork, and positive communication are among the skills such organizations value most to achieve their goals. In this course, you will learn about team culture, strategies for working as part of a diverse team, and techniques for developing a positive, collaborative inclusive mindset.
- *Course 3: Countless Worlds in our Solar System: Asteroids, Comets, and Meteorites:* Our solar system is made up of much more than the Sun and the major planets and their moons. Other small objects, including asteroids and comets, along with the pieces of them that have fallen to the Earth as meteorites, provide important insights into the formation of our solar system. In this course you will learn about these objects’ origin, categorization, composition, location, and features, along with their importance to understanding the history of the solar system. In addition, you will explore NASA missions, as well as jobs and even hobbies, that gather information about these objects.
- *Course 4: Strategies for Troubleshooting Problems in Everyday Life and Work:* Troubleshooting is a type of problem-solving skill needed by everyone, from students programming a toy robot, to families at home trying to get their garage door to open, to NASA engineers tracking down why their spacecraft has stopped sending data to Earth. If you have ever tried figuring out why something is not behaving as it should (*why won’t my lamp turn on?*), you have had a troubleshooting experience! In this course, you will learn about the five global troubleshooting strategies and explore how to choose and use the best strategy for different kinds of problems you face.

## Appendix I: The Psyche Mission Website

Both the Psyche “badge” (“mission identifier”), in multiple approved file formats and color variations, and the corresponding brand guidelines are available on the mission website (https://psyche.asu.edu/galleries/). The mission team worked with designer Michael Taylor to develop the brand guidelines at the onset of the mission. The guidelines are intended to encourage consistent use of the corresponding fonts, colors, sizes, aspect ratios, and positioning. While brand guidelines are often used only internally in many organizational settings, providing the guidelines on the website ensures consistent use of the mission brand standard.

The mission website (Fig. 11) includes a mission overview section with details about the target asteroid, the spacecraft, the launch, the instruments and science investigations, the team, and frequently asked questions (in English and Spanish); the mission timeline; an overview of the relevant science and mission goals and objectives; a news section with articles and blogs written by or about the mission; a list of future and past events; and an image and video gallery. The “Get Involved” section (https://psyche.asu.edu/get-involved/) highlights the offerings of Psyche Student Collaborations, including a public arts opportunity (based on previous NASA efforts such as #JWSTArt and Cassini Inspires), known as #PsycheSpaceCRAFTY (https://psyche.asu.edu/get-involved/psyche-space-crafty/). There is also a page with downloadable or 3D printable models and activities and educational resources (https://psyche.asu.edu/get-involved/models-and-materials/), and links to opportunities to get involved with NASA and mission partners. To make relevant content easier to find in search engines, the Student Collaborations team has implemented search engine optimization (SEO) best practices (keyphrases, meta descriptions, etc.) and a date on each page lets users know the last time content was updated.

As the mission nears major milestones such as launch, Mars gravity assist, orbit insertion, and data collection, the websites at NASA, JPL, and ASU are augmented and expanded to meet communications and public engagement needs. In particular, post-launch the mission website gallery is updated to accommodate processed and calibrated Imager data (see Sect. 5.3.4. Multispectral Imagers Data Direct to Public Websites).

## Appendix J: Mission Social Media (@MissionToPsyche) Brand Strategy and Engagement

The Psyche mission’s social media was initiated in 2016, nearly a year before NASA’s final selection of the mission. The accounts were managed under the @NASAPsyche username across Twitter, Facebook, and Instagram until a rebranding in June 2020 as part of an agency-wide NASA effort to consolidate individual mission social media pages under larger recognizable umbrella accounts. @MissionToPsyche was adopted, along with the general hashtag #MissionToPsyche, in order to post daily about Psyche team featurettes, daily mission insights, student collaboration highlights, and public outreach programs. As noted above, @NASASolarSystem is the designated umbrella account and posts noteworthy mission milestones as well as partnering with @MissionToPsyche to amplify public opportunities when appropriate. All content on @MissionToPsyche social media accounts is organic only, following NASA guidelines.

In October 2019, the team revamped the mission’s social media strategy after identifying three key digital content pillars to focus on: mission and Psyche science-related content (that is not related to mission milestones), student-related content, and public engagement and events. The social media strategy works to incorporate all three elements in the content creation process in a cohesive way to underscore the multifaceted and engaging nature of the mission. From initial selection, the team worked to nurture public interest and encourage involvement through employing a consistent “voice” intended to be welcoming, inspiring, and often playful, employing emojis, puns, relatable GIFs, and friendly responses to mentions. In particular, community management is central to @MissionToPsyche’s digital strategy. The social media team receives a number of social media comments and queries each day and ensures that all responses are equally thoughtful and scientifically accurate by cross-checking with internal and external teams.

@MissionToPsyche has engaged the public through various digital campaigns, with the most prominent campaigns being #PsycheSpaceCRAFTY, #PetsofPsyche, and #PsychePartners. Similar to the Psyche Inspired internship program (see Sect. 2.3), #PsycheSpaceCRAFTY encourages the public to participate in creating and submitting original work inspired by the mission. #PetsofPsyche, developed during the start of the COVID-19 pandemic, aims to share photos of Psyche team members’ pets through global quarantine and beyond. #PsychePartners shares the contributions of the major mission partners (institutions, universities, and other contractors) through their research, engineering, and manufacturing.

### *Instagram Theme Experiment*

A particular benefit of having the accounts managed by a Psyche outreach intern majoring in marketing or communications is that the student is concurrently taking coursework focused on best practices in engaging digital audiences. As an example, originally the Instagram account (which, with 64% of followers reporting as 34 years old or younger, skews younger than the Twitter and Facebook accounts) posted content in the same form as shared on the mission Twitter and Facebook accounts, which left the account with a lack of uniform visible branding and consistent color scheme, important on a visual platform like Instagram. Additionally, complex concepts such as data models proved difficult to interpret without reading accompanying text, which is structured differently on Instagram compared to Twitter or Facebook. Guided by best practices that the social media student interns have learned in their courses, in January 2021 the social media team developed and implemented a “theme” with content-specific templates to give the @MissionToPsyche Instagram page a consistent, cohesive, brand-aligned appearance (Fig. 12). The content was changed to follow a minimal, streamlined standard on a white frame template with a stronger concentration on imagery than text in the photo to appeal to the average user and consistent mission identifier integration.

The theme and associated templates align with the overall Psyche brand guidelines and the appearance of the website, using the mission colors along with a minimalistic white aesthetic (see Sect. 4.1). The content-specific templates (for example, one style for Psyche Inspired artwork, another for upcoming events, and a distinct layout for science and engineering content) were integrated to encourage follower engagement and retention of content by indicating to them, at a glance, what content they can expect from a given post. In the first thirty days of implementing the new visual design, @MissionToPsyche experienced a 300% increase in Instagram Story interactions, 150% increase in website clicks, an almost 40% increase in impressions, and a more than 35% increase in content interactions. The three months following the implementation of theme and its templates resulted in almost double the increase in followers compared to the preceding three months.

### *Expanded Social Media Opportunities*

The mission’s @MissionToPsyche social media accounts (existing, and expanded platforms such as Soundcloud, Pinterest, Twitch, etc.) continue to provide day-to-day mission and Student Collaborations content, highlight opportunities to get involved, and share an inside look at the team. Surrounding launch and post-launch, as mission milestones and newsworthy events increase, @NASASolarSystem accounts are designed to feature Psyche more frequently and also begin to implement special social media opportunities such as NASA Socials, NASA Live content, social media features, and other activities. Additionally, as in the past, there will continue to be efforts to highlight various Psyche team members in their roles pre- and post-launch through open question-and-answer formats and interviews. This provides a closer look into the behind-the-scenes of Psyche and allows members of the public to pose questions to mission experts. In October 2019, the Psyche outreach interns coordinated Psyche’s first Reddit “Ask Me Anything” (AMA), which was verified and cross-posted to the subreddit r/space with over 15 million active members, and Ernest Cisneros, Psyche’s Science Data Center Manager, fielded questions from the public. Similarly, in December 2020, Dr. David Williams, science team co-investigator held a live Zoom Q&A and in March 2021, NASA’s Jet Propulsion Laboratory led a Facebook Live Q&A with project systems engineer David Oh from inside High Bay 1, showcasing the newly delivered Psyche spacecraft in the background.

Another objective for the Psyche team is to increase participation across emerging, approved platforms, such as Twitch and Clubhouse. With the goal of making the mission appealing and accessible to as many people as possible, the mission plans to contribute to a multi-channel approach in expanding the mission’s messaging, public education, and outreach. NASA currently operates an official Twitch account with over 900,000 followers, where it highlights rocket launches, spacewalks, and other major occasions. In the future, Psyche hopes to help highlight team members, public engagement events, and NASA livestreams related to pre- and post-launch milestones to share with the public. Additionally, Clubhouse presents as a unique opportunity for the Psyche mission and its team to host informative virtual panels and events; Psyche’s principal investigator was a guest speaker at a space-themed Clubhouse event in March 2021. The team seeks to build and grow followings on emerging platforms such as these to reach new audiences and spread the mission’s message.

Finally, Psyche’s current Student Collaborations alumni program (Sect. 5.3.3) includes many hundreds of current students and graduates from institutions across the United States. Through LinkedIn, the messaging app Slack, and a periodic email newsletter, the mission remains connected to program alumni by sharing mission updates, opportunities to stay involved, and relevant job posts.

## Appendix K: Psyche Mission Videos

### *Mission Trailer Video*

The mission trailer video, “Journey to a Metal World” (https://www.youtube.com/watch?v=aExTQGcIGKo&t=1s) was launched in 2018 and filmed at Arizona State University and at NASA’s Jet Propulsion Laboratory (with an update released in early 2022). In addition to the principal investigator, Lindy Elkins-Tanton and project manager Henry Stone, the video features Psyche team engineers, scientists, students, and staff. Throughout this five-minute award-winning video (2018 Rocky Mountain Emmy Award for Best Director, Short Form), which airs on the NASA YouTube channel, viewers are taken on a journey to explore why this mission was selected for NASA’s Discovery Program, how the spacecraft will get to the asteroid, what the team hopes to learn from Psyche, and the importance of scientific discovery. It includes a call to action for the public to “Join the Journey” via the NASA and Psyche websites. The link to this video is shared with all incoming Student Collaborations participants as part of their onboarding process.

### *Instrument and Investigations Highlight Videos*

The Psyche team produces video vignettes on each of Psyche’s instruments, the Gamma Ray and Neutron Spectrometer (GRNS), the Magnetometer, and the Multispectral Imager, as well as a video featuring the gravity science investigation. Other videos related to technology (such as the Deep Space Optical Communications [DSOC] technology experiment), engineering, or science may also be undertaken throughout the mission. The goal of the videos is to introduce each instrument or investigation, its purpose on the mission from a science and engineering perspective, and the team members involved. As with the Mission Trailer, the instrument videos tell the story of the mission and the teams involved for a general audience.

### *Animated Mission Videos*

In addition to interviews with team members about various aspects of the mission, several animated videos were developed to illustrate the arrival of the spacecraft at the asteroid, to help explain the asteroid’s potential violent past, and to simulate what a metallic world might look like based on the latest research. Artist and cinematic illustrator Peter Rubin created these videos in a series which includes an introduction to the mission with a voice over by mission principal investigator Linda Elkins-Tanton (https://www.youtube.com/watch?v=cgtiwqVWfgc), an illustrated video of Psyche’s story and the violent impacts that may have stripped away its mantle (https://psyche.asu.edu/galleries/videos/), and an animation of the Psyche spacecraft exploring and charting the metallic world.

### *Science and Engineering Team Highlights Videos*

Throughout the mission, science and engineering team members are selected for video features to highlight their role on the mission, their background, and their area of expertise. To date, science team members Dr. Simone Marchi from the Southwest Research Institute and Dr. Tim McCoy from the Smithsonian Museum of Natural History have been featured. Additional science and engineering team members will be featured as the mission progresses, both by the Psyche mission and by the NASA 360 crew, to showcase the science behind the mission and the breadth of skills and abilities needed to compile a successful space mission team.

### *Student Collaborations Videos*

For promotion to and recruitment of future participants and stakeholders, short videos were made about the Psyche capstone program (https://psyche.asu.edu/gallery/nasa-psyche-mission-undergraduate-capstone-projects/) and the Innovation Toolkit Online Courses (https://psyche.asu.edu/gallery/asu-psyche-online-course/) by the Psyche mission website design team, Fervor Creative. The Capstone program video features testimonials from Psyche capstone alumni and showcases a number of their projects as well as explaining for potential capstone faculty or students the ways to get involved. The Innovation Toolkit Online Courses video emphasizes the opportunity to join the courses at any time from any place and encourages lifelong learners worldwide to build their skills for innovation and exploration.

Videos created by Student Collaborations participants themselves (outreach interns, capstone students, and Psyche Inspired interns) augment the content for the website, social media, and school visits and other outreach events. For example, in response to a request from 4th grade teachers during a school visit, a group of outreach interns researched, wrote the script and had it approved by the Psyche team, recorded, and animated a two-part video series about the engineering process: (https://psyche.asu.edu/2020/12/04/engineering-process-part-1/; https://psyche.asu.edu/2020/02/07/engineering-process-part-2/). Capstone students have made quick videos about Psyche for posting on #PsycheSpaceCRAFTY (e.g., https://psyche.asu.edu/psyche-space-crafty/christina-webb-stephanie-bookout/) and Psyche Inspired interns frequently create videos as a means of capturing their work (https://psyche.asu.edu/gallery/sixteen/) or sharing the mission through animation and song (https://psyche.asu.edu/gallery/an-asteroid-named-psyche-animated-typography/). They are also interviewed for their annual showcase and relevant excerpts from those interviews are compiled into videos used for promoting the annual Psyche Inspired application (e.g., https://www.youtube.com/watch?v=nr2DEmzeSuw).
